# Systematic NMR Analysis of Stable Isotope Labeled Metabolite Mixtures in Plant and Animal Systems: Coarse Grained Views of Metabolic Pathways

**DOI:** 10.1371/journal.pone.0003805

**Published:** 2008-11-25

**Authors:** Eisuke Chikayama, Michitaka Suto, Takashi Nishihara, Kazuo Shinozaki, Takashi Hirayama, Jun Kikuchi

**Affiliations:** 1 RIKEN Plant Science Center, Yokohama, Kanagawa, Japan; 2 Yokohama City University, Yokohama, Kanagawa, Japan; 3 Nagoya University, Nagoya-shi, Nagoya, Aichi, Japan; University of Florence, Italy

## Abstract

**Background:**

Metabolic phenotyping has become an important ‘bird's-eye-view’ technology which can be applied to higher organisms, such as model plant and animal systems in the post-genomics and proteomics era. Although genotyping technology has expanded greatly over the past decade, metabolic phenotyping has languished due to the difficulty of ‘top-down’ chemical analyses. Here, we describe a systematic NMR methodology for stable isotope-labeling and analysis of metabolite mixtures in plant and animal systems.

**Methodology/Principal Findings:**

The analysis method includes a stable isotope labeling technique for use in living organisms; a systematic method for simultaneously identifying a large number of metabolites by using a newly developed HSQC-based metabolite chemical shift database combined with heteronuclear multidimensional NMR spectroscopy; Principal Components Analysis; and a visualization method using a coarse-grained overview of the metabolic system. The database contains more than 1000 ^1^H and ^13^C chemical shifts corresponding to 142 metabolites measured under identical physicochemical conditions. Using the stable isotope labeling technique in *Arabidopsis* T87 cultured cells and *Bombyx mori*, we systematically detected >450 HSQC peaks in each ^13^C-HSQC spectrum derived from model plant, *Arabidopsis* T87 cultured cells and the invertebrate animal model *Bombyx mori*. Furthermore, for the first time, efficient ^13^C labeling has allowed reliable signal assignment using analytical separation techniques such as 3D HCCH-COSY spectra in higher organism extracts.

**Conclusions/Significance:**

Overall physiological changes could be detected and categorized in relation to a critical developmental phase change in *B. mori* by coarse-grained representations in which the organization of metabolic pathways related to a specific developmental phase was visualized on the basis of constituent changes of 56 identified metabolites. Based on the observed intensities of ^13^C atoms of given metabolites on development-dependent changes in the 56 identified ^13^C-HSQC signals, we have determined the changes in metabolic networks that are associated with energy and nitrogen metabolism.

## Introduction

The tremendous advances in DNA sequencing technologies that have taken place over the last few years have made large-scale sequencing and genome comparison studies accessible for many applications. For example, the cost of human genotype characterization is now about $1000 per genome [1]. These technologies have also made comparative metagenomic analyses of complex microbial communities sampled from diverse environments possible [2]. However, methods are still needed to tie function to sequence, a step that is necessary to efficiently identify proteins that might serve as drug targets [3], or enzymatic activities that could be used to produce valuable products such as biofuels or other biomaterials [4]. Because cellular metabolic contents or byproducts are good indicators of both genetic and environmental factors, metabolic phenotyping has found a place in the post-genomics and proteomics era [5], [6]. Therefore, technological developments in both nuclear magnetic resonance (NMR) and mass spectrometry (MS) have provided key insights into metabolic phenotypes over the last half decade [7], [8]. Versatile sampling and robust ^1^H-NMR methods have improved disease diagnosis [9]–[12] and personalized healthcare [13]–[15], and have provided an important tool for the characterization of genetic variations [16]. The use of high-resolution magic angle spinning methods has had an especially beneficial effect on measurements in intact tissues and in whole organisms [17]–[19].

The introduction of stable isotope (SI) labeling techniques [20] has turned out to be an essential development for NMR analyses of metabolism. SI labeling-based metabolic analyses have been widely used in bacterial systems [21], and in higher plants and animals [22], [23]. Metabolic flux analysis is a powerful method for following an incorporated ^13^C, or ^2^H nucleus through specific metabolic pathways in mammalian systems [24]–[26]. Furthermore, recent increases in the sensitivity of NMR detection with a dynamic nuclear polarization technique combined with *in vivo* ^13^C labeling have allowed metabolic imaging assays to be completed on a much faster time scale and with finer spatial resolution [27], [28].

Although these methodological advances have widely contributed to metabolic analyses in plant and animal systems, a key component of the overall technology is the accurate identification of metabolites in unpurified samples, including *in vivo* measurements. Using comprehensive SI labeling of metabolites in plants, we have recently shown that heteronuclear single quantum coherence (HSQC)–based 2D-NMR [29] enables higher resolution and sensitivity than classical 1D-NMR–based approaches [30]–[32]. The use of comprehensive SI labeling results in better signal-to-noise ratios for metabolites present in low concentrations, and makes ^13^C–^13^C through-bond connectivity experiments possible (*i.e.* HCACO) [33]. Another strategy for identifying metabolite signals in crude biological mixtures involves the use of a standardized chemical shift metabolomics database [34], [35]. This sort of database can only be reliably used if the quality of the datasets is assured by eliminating chemical shift data that have not been generated in a standard buffer and at a constant temperature. For example, the chemical shift data in conventional public [36] or commercial databases are inadequate for NMR-based metabolome studies because they were compiled with the main goal being to collect information on the largest possible number of compounds, but were measured under different solvent, temperature, pH, and ionic strength conditions, resulting in the potential for significant identification errors. The use of SI-labeled metabolites and an HSQC-based chemical shift database would allow the comprehensive detection of large numbers of metabolites in a metabolic network study.

SI-based metabolic flux analyses have been used to identify metabolic networks at the atomic level [24]–[26]. However, this ‘bottom-up’ approach [37] could be complemented by the analysis of macroscopic systems such as biological fluids, cells, or even tissues. This sort of ‘top-down’ metabolite analysis, or metabolic phenotyping, currently employs Principal Components Analysis (PCA) or its variations. As a result, complicated metabolic systems can be visualized by ‘birds-eye-view’ technology with quantitative multivariate calculation, whereas previous “bottom-up” approaches employ a focused, microscopic view of specific reactions and molecules. These “top-down” methods, and presumably others in various stages of development, can and will be employed in higher systems-level approaches. For example, invertebrate models are versatile systems which can be used for chemical phenotyping applications, such as drug discovery [38]. Compared with bacterial or animal cell-line assays, invertebrate-based or fish-based assays can be used to identify physiological changes at the tissue or organ level, and can be used for large-scale metabolic screening of bioactive compounds [39], [40] to provide a coarse-grained view of complicated biological networks [41]–[43].

In this report, the concept of systematic NMR analysis of stable isotope-labeled metabolite mixtures is described and tested. Our concept consists of SI labeling of living plants and animals, and systematic metabolite identification using an HSQC-based metabolite chemical shift database combined with 2D and 3D NMR. We applied these methods to the metabolic analysis of two higher organisms whose genomes have been sequenced, *Arabidopsis* T87 cultured cells, and the model invertebrate, *Bombyx mori* (silkworm) larvae [44], [45], both of which have been used for studying physiological responses to pathogens and for screening potential human drugs by observing their phenotypes [46]. An additional goal was to devise a visual representation of silkworm metabolic organization during a major developmental transformation by analyzing changes in ^13^C intensities of metabolites in a pathway. A time-series NMR analysis of ^13^C-labeled metabolites was used to represent metabolic changes as a coarse-grained view of KEGG reference metabolic pathways [47].

## Results

### HSQC-based metabolite chemical shift database

**Figure 1:**
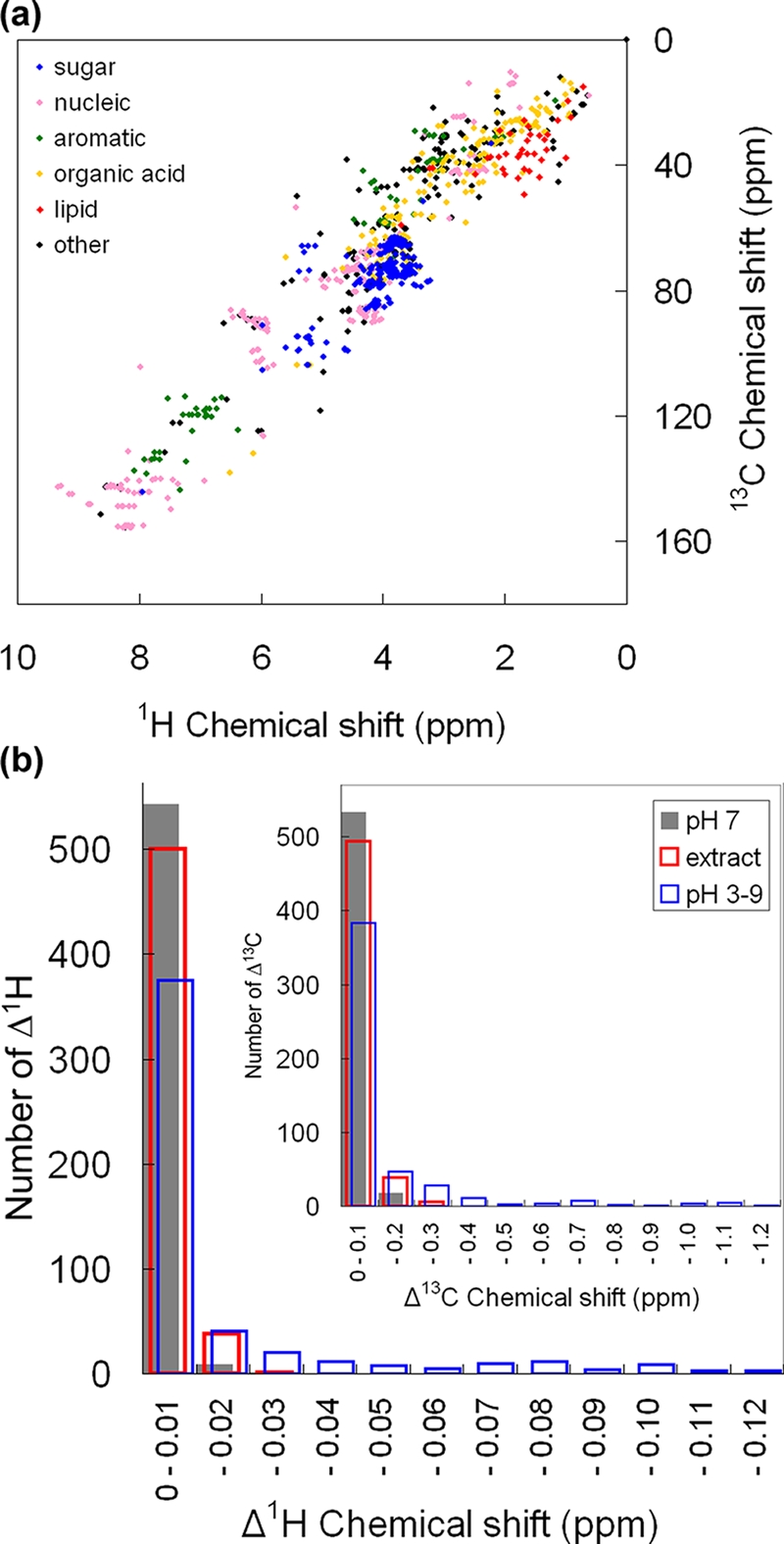
HSQC-based metabolite chemical shift database. (a) 1018 HSQC peaks derived from 142 metabolites compiled in the database are displayed as sugars (blue), nucleic acids (pink), metabolites having aromatic rings (green), organic acids (orange), lipids (red), and other metabolites (black). (b) The robustness of the database was tested statistically in terms of both ^1^H and ^13^C (upper right) chemical shifts (Δ^1^H and Δ^13^C, respectively). Chemical shift fluctuations of reference compounds (16 proteinogenic amino acids, glucose, succinate, and fumarate) with T87 crude extracts (red, 539 chemical shifts) were small enough to be used as representative chemical shifts for comparison with database records. Chemical shifts of reference compounds without added extracts are shown in gray (553 chemical shifts). In contrast, the chemical shifts of reference compounds at different pHs (blue, 525 chemical shifts) fluctuated over a wider range (compare blue and gray bars). A chemical shift fluctuation was defined as the difference from the average of the chemical shifts without added extracts.

An HSQC-based metabolite chemical shift database was compiled that contains only NMR spectra of standard compounds measured under standardized temperature, pH, solvent, and ionic strength conditions. The database thus contains only highly reproducible chemical shifts, which makes it a reliable source for identifying metabolic products. Currently it consists of 1018 ^1^H and ^13^C chemical shifts corresponding to 142 metabolites. Each chemical shift was derived from an NMR spectrum for one or a few metabolites measured at 298 K and dissolved in 100 mM potassium phosphate buffer at pH 7.0. Chemical shifts were distributed from 0.6 to 9.4 ppm for ^1^H and from 10.4 to 155.7 ppm for ^13^C (Fig. 1a). The average number of ^13^C-HSQC peaks per metabolite was 7.2. A linear regression of all 1018 peaks indicated that a ^13^C chemical shift = 17.5×^1^H chemical shift – 2.5 (*r* ^2^ = 0.892). The ^1^H and ^13^C chemical shifts were around 2 and 40 ppm in lipids, 3.5 and 70 ppm in sugars, and 7 and 130 ppm in aromatic compounds, respectively. There were dense and sparse regions along the regression line, with an especially dense region near 3.5 ppm of the ^1^H shift, due mostly to sugars. On average, however, 58% of the ^13^C-HSQC-derived chemical shifts per metabolite did not overlap with any other metabolites in the database if the tolerances for overlapping were set at 0.03 and 0.3 ppm for ^1^H and ^13^C chemical shifts, respectively. As a result, 130 (92%) of 142 metabolites included one or more chemical shift markers, which we defined as chemical shifts that did not overlap with any other metabolites in the database.

The robustness of the HSQC-based metabolite chemical shift database was tested with actual biological extracts. Standard reference compounds were dissolved in the standardized buffer with and without added crude *Arabidopsis* T87 cell extracts and the chemical shifts of the two solutions were compared. Sixteen proteinogenic amino acids (16AA, excluding asparagine, glutamine, cysteine, and tryptophan) along with succinate, fumarate, and glucose were used as reference compounds. The distribution of chemical shift fluctuations (*n* = 7, corresponding to more than 500 chemical shifts) in the solution with T87 extracts were all within 0.03 ppm for both ^1^H and ^13^C (**Fig. 1b**, red), which is comparable to the experimental resolution (0.01 and 0.5 ppm for ^1^H and ^13^C, respectively).

None of the samples fluctuated in pH (average 7.2; standard deviation<0.1). Reference compound chemical shift fluctuations in standardized buffer (pH 7) and in buffers from pH 3 to pH 9 were used as controls (**Fig. 1b**). There was a broad distribution of chemical shift fluctuations in buffer solutions with a pH 3 to 7, though 83.1% were still within 0.03 ppm. For example, the chemical shifts of phenylalanine C_β_, arginine C_δ_, and proline C_δ_ showed little pH dependence, but glycine C_α_ and histidine C_β_ shifts were relatively pH-dependent (**Fig. S1**).

### Systematic batch identification of metabolites

**Figure 2:**
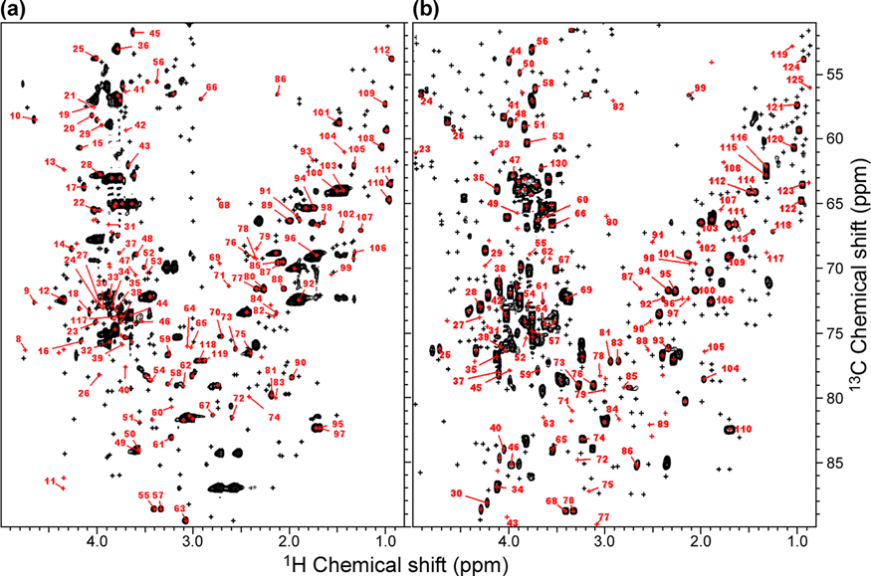
Metabolite identification using the HSQC-based metabolite chemical shift database. (a) A total of 453 peaks were detected in the *B. mori* HSQC spectrum, of which 174 had candidate matches in the database (red), and 119 were uniquely identified (no more than one candidate match, numbered in red; see Table S1 for the metabolite names), and 279 had no candidate matches (black). The spectrum was acquired with 2048 points per 16 ppm of ^1^H, 32 transients per free induction decay, and 160 increments per 40 ppm of ^13^C. (b) A total of 544 peaks were detected in extracts from T87 cultured cells, of which 192 had candidate matches in the database (red) and 124 were uniquely identified (numbered in red; see Table S1), and 352 had no candidate matches (black). The spectrum was acquired with 1024 points per 16 ppm of ^1^H, 32 transients per free induction decay, and 200 increments per 40 ppm of ^13^C.

An SI-labeling technique, the HSQC-based metabolite chemical shift database, and heteronuclear multidimensional NMR spectroscopy were combined in a systematic method for simultaneously identifying a large number of metabolites. ^13^C-HSQC spectra of crude silkworm and T87 cell extracts were analyzed with an in-house program that consults the database and compares ^13^C-HSQC spectra with the data. A large number of metabolites were identified in a single batch experiment (**Fig. 2**). However, although this system can only be used to identify candidate metabolites by matching observed chemical shifts with those in the database, it is capable of high-throughput, which makes large-scale approaches possible. In the silkworm extract, 453 peaks appeared in the ^13^C-HSQC spectrum (**Fig. 2a**). 174 of them had candidate matches in the database, corresponding to 95 metabolites, and 119 of the 174 peaks, corresponding to 57 metabolites, had a unique candidate match. The most abundant identified metabolite by peak intensities was lysine, which is a hub metabolite [48]. Other abundant metabolites were the hub metabolite glycine, and glutamine. Further analysis using 3D HCCH-COSY [49] (data not shown) also detected cross peaks corresponding to trehalose, glucose, lysine, glutamine, alanine, proline, threonine, leucine, ornithine, and 4-hydroxy proline. The 57 uniquely identified metabolites are related to 81 metabolic pathways, according to the Kyoto Encyclopedia of Genes and Genomes (KEGG) database [47]. There were 544 ^13^C-HSQC spectrum peaks in the T87 cultured cell extract, of which 192 correspond to 108 metabolites; and 124 of the 192 peaks, corresponding to 61 metabolites, were uniquely identified in the database (**Fig. 2b**). These 61 metabolites are related to 73 metabolic pathways. The most abundant identified metabolite was choline, and other abundant metabolites were d-gluconic lactone, glycerol, gamma-aminobutyric acid (GABA), lysine, uridine, and glucose. Lysine and glucose were abundant in both silkworm and T87 extracts, whereas other metabolites were abundant in one or the other but not both. Peak intensities ranged from 1 to 10^−3^ or 10^−4^ in both silkworm and T87 cells, and we observed a power-law relationship between the number of peaks and peak intensities [50]–[52]; that is, the majority of metabolites were present in lower concentrations, whereas a minority of metabolites were at higher concentrations (**Fig. S2**).

**Figure 3:**
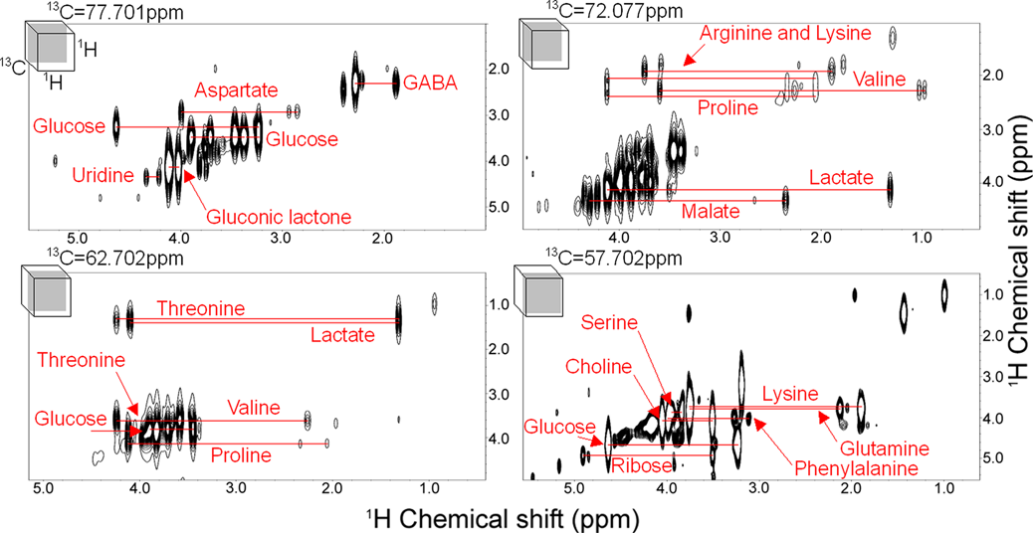
3D HCCH-COSY spectrum. A heteronuclear 3D NMR spectrum of crude ^13^C-labeled T87 cultured cell extracts. 2D ^1^H–^1^H planes at 57.702, 62.702, 72.077, and 77.701 ppm of ^13^C cut through the 3D HCCH-COSY spectrum are shown (for each spectrum, the relative position of each cut is shown in a 3D box at the upper left corner). Red lines connect detected cross peaks of the named metabolites. In total, 89 HSQC peaks corresponding to 24 metabolites were confirmed. The spectrum was acquired with 1024 points per 14 ppm in the ^1^H direct dimension, accumulating 16 transients per free induction decay, 100 increments per 40 ppm in the ^13^C dimension, and 48 increments per 8 ppm in the indirect ^1^H dimension.

Assignments in which the chemical shift matched more than one candidate metabolite in the database could be refined or avoided by using 3D HCCH-COSY spectra, a 3D heteronuclear NMR experiment in which another dimension is added to 2D (**Fig. 3**). Four ^1^H–^1^H planes cut the 3D ^1^H–^1^H–^13^C spectrum of the T87 extract sample at various points along the ^13^C axis, which allows the identification of ^1^H–^13^C–^13^C–^1^H bonds, thus confirming ^13^C-HSQC assignments by using ^1^H–^13^C–^13^C–^1^H information to connect two ^1^H–^13^C HSQC peaks (**Fig. 2b**). For the first time then, efficient ^13^C labeling provides reliable signal assignment from 3D HCCH-type spectra in higher organism extracts. A number of cross peaks were thus be identified which correspond to 89 HSQC peaks and 24 metabolites. For example, GABA, asparagine, glucose, uridine, lactate, malate, choline, and ribose could be used to verify the identification, thus resulting in a high degree of accuracy (**Table S2**).

### Pilot application: coarse-grained views of overall physiological changes in silkworms fed with ^13^C-labeled nutrient

**Figure 4:**
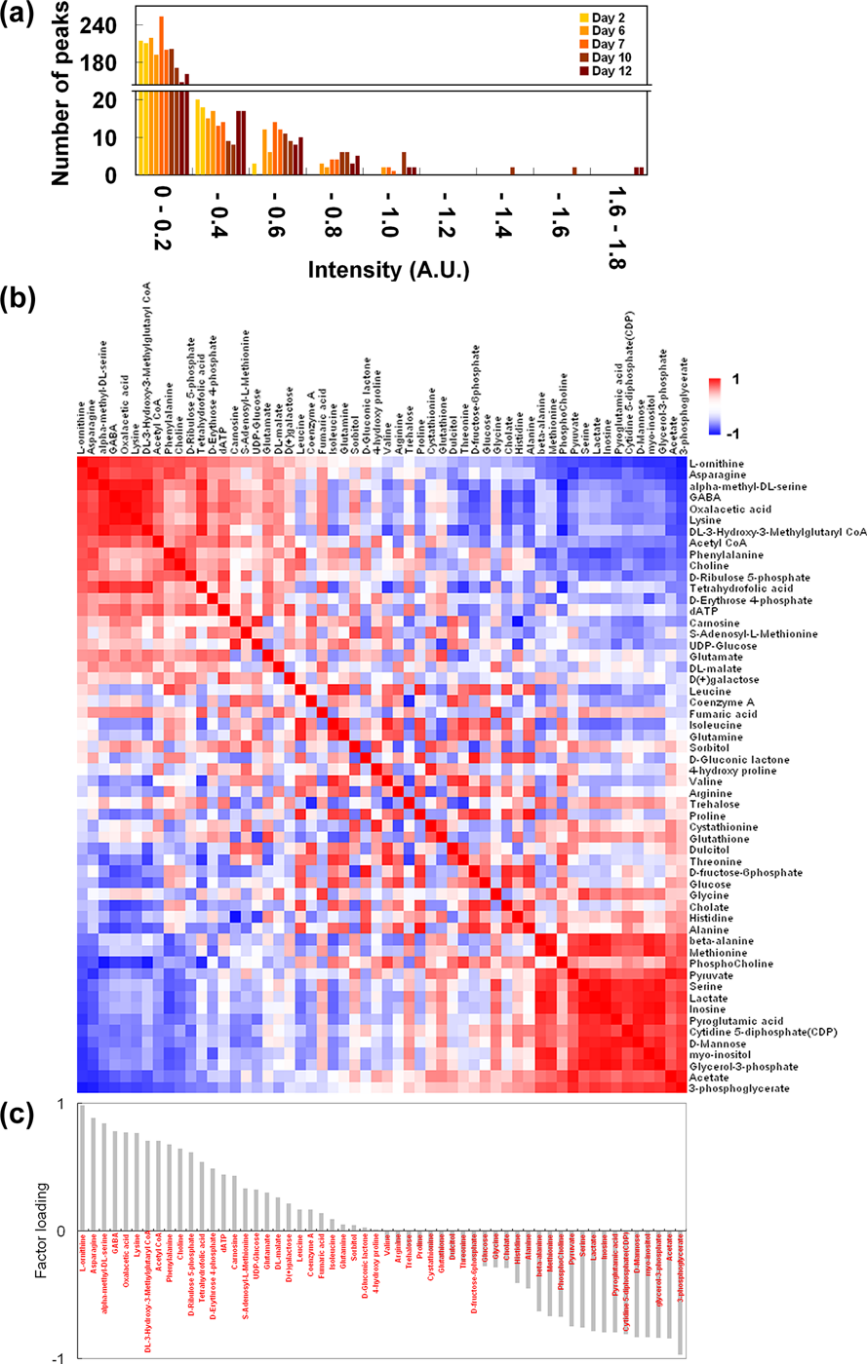
^13^C labeling of *B. mori* larvae as a pilot application in an invertebrate animal model. (a) Fourth instar silkworm larvae were reared on ^13^C-labeled diets for 12 days through ecdysis to the fifth instar stage, resulting in the gradual assimilation of ^13^C-labeled metabolites. Two bars per sample stage are shown. (b) Correlation matrix of 56 commonly selected metabolites used for the analysis of metabolites detected during the 12 days of the feeding experiment. Positively correlated metabolites are in red and negatively correlated metabolites are in blue. The metabolites are sorted by their loadings on PC1. (c) Loadings of the metabolites on the PC1 axis.

**Figure 5:**
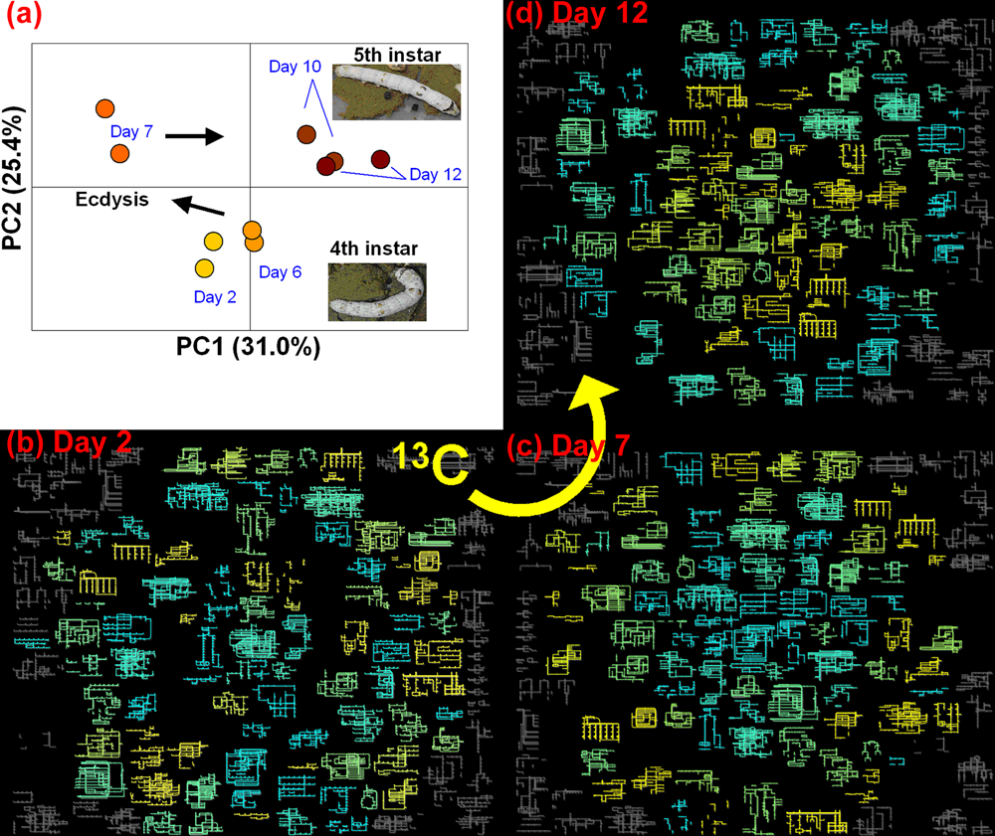
Coarse-grained views of metabolic pathways computed from ^13^C-labeled B. mori during the developmental changes associated with fourth to fifth instars. (a) PCA score plot. The sample stages were classified into fourth instar (days 2 and 6); post ecdysis (day 7); and fifth instar (days 10 and 12). Note that incorporated nutrients are stored toward metamorphosis after the fifth instar stage. (b–d) Coarse-grained representations of silkworm metabolic pathways. The loadings on PC1 of the 56 metabolites were mapped onto all 132 KEGG metabolic pathways as total loadings and the positions of the pathways in a plane were calculated by a simulated annealing technique with total intensities. Total loadings are represented by yellow, cyan, or their intermediates, by linearly superimposing colors corresponding to the loadings of metabolites included in each pathway. Yellow (or cyan) pathways have mostly positive (or negative) metabolites in loading on PC1, and green pathways result from the linear mixture of yellow and cyan. Gray pathways contained no identified metabolites. The simulated annealing technique used the total intensities at each sampling stage rather than total loadings, which optimized the locations of pathways with larger (or smaller) total intensities toward the center (outside). Movement of the metabolic state from negative to positive on the PC1 axis, caused a coincident movement of yellow (cyan) pathways, reflecting actively accumulating (dispersing) ^13^C atoms. The plot may include implicit coarse-grained information about the metabolic system that includes higher-dimensional correlations, or organization, among metabolic pathways.

A pilot ^13^C labeling experiment was conducted to test whether our method could be used to visualize the metabolic state of a model invertebrate. *B. mori* larvae were fed an SI-labeled diet containing [^13^C_6_]glucose and [^13^C,^15^N]amino acids for 12 days, a time which corresponds to the larval developmental course that extends from the fourth instar through ecdysis into the fifth instar. Extracts were sampled on days 2, 6, 7, 10, and 12. Duplicate ^13^C-HSQC spectra were recorded on each of the five sampling days to detect and identify metabolites. Peak intensities in each spectrum gradually increased throughout the experiment, demonstrating that ^13^C atoms accumulated in the larval bodies (**Fig. 4a**). There were in all 56 different candidate metabolite matches that could be used to follow metabolic changes (**Table S3**). Of the 56 candidate metabolites, 71% were also found in the silkworm batch experiment (compare **Tables S1** and **S3**). The total peak intensities of all 56 candidate metabolites in relation to sampling days (**Fig. S3**) were converted to a 56×56 (metabolite–metabolite) correlation matrix (**Fig. 4b**). The matrix was analyzed by principal components analysis (PCA) and PC loadings were determined (**Fig. 4c** for PC1). The matrix showed clear positive and negative correlations in peak intensities among the 56 metabolites. For example, l-ornithine (**Fig. 4b**, first row) was positively correlated with asparagine (second row) but negatively correlated with 3-phosphoglycerate (last row). These correlations were interpreted as indicators of physiological changes that accompany the developmental progress of silkworm larvae. On the basis of the first two principal components (PC1 and PC2), larval growth was divided into three phases: the fourth instar (days 2 and 6), just after ecdysis (day 7), and the fifth instar (days 10 and 12, **Fig. 5a**). The contribution ratios were 31.0% by PC1 and 25.4% by PC2. We selected the PC1 as the better indicator of metabolic change from day 7 to day 12. Since PC1 loadings (**Fig. 4c**) correspond to the metabolic change, all 132 KEGG reference metabolic pathways were mapped to obtain a coarse-grained view of the metabolic system of a silkworm at each of three sampling stages (**Fig. 5b–d**). Each pathway could then be assigned a defined total loading and a total intensity. Total loading was defined as the sum of the loadings on PC1 of the detected metabolites included in a pathway. Similarly, total intensity was defined as the sum of the standardized intensities of the detected metabolites in a pathway (see **Fig. S3** for intensities before standardization). Total loadings and total intensities can be represented as colors and positions, respectively, so the same pathway retains the same color throughout the time course. Positions were determined with a simulated annealing technique by optimizing pathways that had large total intensities toward the center and those having small total or null intensities away from the center (**Fig. 5b–d**). By comparing the patterns in color and position, we found that the positions of the pathways were differently laid out at each sampling stage. The coarse-grained pattern of metabolic pathways at day 2 (**Fig. 5b**) appears to be random because it was not highly correlated with metabolic changes along the PC1 axis. In contrast, days 7 and 12 show clear patterns, indicating the emergence of organization among the labeled metabolic pathways (**Fig. 5c and d**). Pathways that were negatively correlated with the PC1 axis (cyan) crowded the center of the figure at day 7 (**Fig. 5c**), but migrated away from the center by day 12 (**Fig. 5d**). Similarly, pathways that were positively correlated with the PC1 axis (yellow) were peripheral at day 7 (**Fig. 5c**), but crowded the center by day 12 (**Fig. 5d**). This observation offers a coarse-grained view of metabolic changes taking place along the PC1 axis, and suggests that the roles of some pathways interchange along the PC1 axis during larval development. The pathways in the KEGG database with the most positive and most negative total loadings were glutamate metabolism (contributed from GABA, glutamate, malate, oxalacetate, fumarate) and glycolysis/gluconeogenesis (contributed from 3-phosphoglycerate, lactate, and pyruvate), respectively (see **Table S4**). From the point of view of PC1 metabolite loadings, pathways could be classified into two categories: those including detected metabolites of mostly positive PC1 loadings (yellow) and those of mostly negative ones (cyan). Furthermore, contributions from positive and negative loadings in the same pathway appear as gradations of green (see **Text S1** for specific methods used to make these calculations). Observed ^13^C intensities for metabolites in a given pathway can thus be viewed averagely as yellow for almost metabolites increasing in terms of ^13^C, cyan for almost decreasing ones, and greenish intermediate colors (*i.e.* combinations of yellow and cyan) for combinations of increasing and decreasing ones. These coarse-grained representations allow visualization of the emergence of organization among cooperating pathways starting around day 7 and reversed by day 12. Conversely, there was little evidence for pathway organization at day 2. Related pathways could be expected to have correlated changes during development or other coordinated metabolic changes. For example, glutamate metabolism, the urea cycle, and metabolism of amino groups, which have positive total loadings, are possibly correlated. Similarly, glycolysis/ gluconeogenesis and cysteine metabolism, which have negative total loadings, should also be correlated (**Table S4**).

## Discussion

Our method of systematic metabolite identification using an HSQC-based metabolite chemical shift database is practical. Because of the tremendous number of both primary and secondary metabolites in nature, a highly efficient strategy for accumulating chemical shift data for many biological compounds is required for high-throughput NMR-based metabolic analyses. The HSQC-based database is a fast and cost-effective way of accumulating information about a huge number of standard compounds, although further specific analyses of NMR-based atomic assignments using NMR spectra obtained by additional methods such as COSY or total correlation spectroscopy (TOCSY) are required. The requirement that HSQC spectra be measured in a standardized buffer under physicochemically constant conditions is critical for minimizing identification errors. In general, crude extracts contain a mixed bag of biological molecules such as metabolites, ions, and biopolymers. Although these compounds have the potential to cause unpredictable fluctuations in the chemical shifts of metabolites, all of the reference compounds dissolved in standard buffer with crude T87 extracts had fluctuations within 0.03 ppm for both ^1^H and ^13^C (**Fig. 1b**, red). This suggests that identifying metabolites in (ideally dilute) crude extracts by using only standardized representative chemical shifts is generally practical for most metabolites, although the chemical shifts of some metabolites tend to fluctuate slightly. Citrate, which has been discussed elsewhere [53], is one such example (data not shown). In our experiment on pH dependence (**Fig. 1b**, blue), more than 80% of fluctuations were small, also indicating that metabolite identification with our method is reasonable for *in vivo* NMR experiments.

Our HSQC-based detection method (**Fig. 2**) offers more reliable metabolite identification than detection based simply on ^1^H 1D NMR spectra, which are frequently used in NMR-based studies on drug toxicity and gene function [6]. A previous report described a ^1^H-NMR-based method which was used to identify 53 metabolites, GC/MS was used to identify 40 metabolites, and LC-FT/MS was used to identify 17 [54]. Recent reports using ^1^H-NMR-based methods indicate that somewhere between 40 to 60 metabolites can be identified [55]–[58], but ^1^H-NMR-based methodology has its limits [54]. The introduction of ^13^C nuclei offers more accurate metabolite identification, but does not necessarily increase the total number of identifiable compounds [59]–[61]. It is likely, however, that expansion in the contents of the database will improve this situation.

One apparent shortcoming of our method, however, is false positives, which might occur when the number of database records or user query peaks is very large. This is because there are more metabolites when the concentration of each is lower (**Fig. S2** and related studies supporting these results [50]–[52], [62]). A false positive is an observed peak that has been wrongly identified as belonging to a particular metabolite. One solution to this problem is to use defined chemical shift markers. For example, our database includes more than 90% of metabolites that had one or more internal chemical shift markers [*i.e.* those having tolerances of 0.03 (^1^H) and 0.3 ppm (^13^C)]. Even using tolerances of 0.1 (^1^H) and 1.8 ppm (^13^C), which are significantly larger than what is normally used, this calculation reaches 40%, suggesting that, by knowing which peaks are chemical shift markers, peaks can often be used to discriminate between metabolites, greatly helping to preclude false positives. This validation can be accomplished merely by knowing which peaks can be uniquely identified by our definition. Any increase in the content of the defined conditions database improves the reliability and utility of the chemical shift markers. Expanding the database thus increases both the range and accuracy of metabolite identification. We are currently developing a method for predicting candidate compounds from unknown chemical shifts in HSQC-spectra of biological extracts. New methodologies like this one, and empirical tests will likely contribute toward truly comprehensive metabolite identification, and thus overcome the problem of simultaneously identified metabolites.

In the case of molecules, or types of molecules with overlapping chemical shifts (*e.g.*, sugars generally tend to overlap in the database), other techniques will be required. One possible solution is to combine data from our method with those of other 2D NMR experiments, which are used to identify compounds in complex mixtures [59], [60]. Additionally, we demonstrated that the number of ambiguous assignments could be reduced by the introduction of a third dimension, that is, by using heteronuclear 3D NMR spectra (**Fig. 3**). HCCH-COSY spectrum analysis is a good example of the use SI labeling techniques in living organisms, since the natural abundance of ^13^C–^13^C bonds is very low (1/100×1/100 = 1/10000), making them impossible to detect without SI amendment. Currently, heteronuclear 3D or higher-dimensional NMR experiments are standard methods in protein NMR spectroscopy [63], and one point of emphasis in this current work was to test the feasibility of using heteronuclear 3D NMR spectroscopy in NMR-based metabolome analysis. In addition to the problem of false positives, there is a general lack of confidence in the reproducibility of correct metabolite identification in crude biological extracts. In fact, there were frequent fluctuations in the observations of metabolites from individual silkworm extracts (for example, compare **Tables S1** and **S3**). Such fluctuations may be due to individual genetic differences, subtle heterogeneous growth or developmental conditions, or artifacts introduced during sample preparation. Another challenging problem associated with the use of NMR techniques in metabolome analysis is low sensitivity, but some possibilities for resolving these shortcomings have been proposed [64], [65].

Based on a new type of metabolomic analysis, we have presented a method for coarse-grained representation of changes in a metabolic network in developing silkworms (**Fig. 5b–d**), whereas classical SI-labeled metabolic studies are based on targeted analyses of specific metabolic pathways, such as glycolysis or gluconeogenesis. [24]–[26]. Recent metabolomics/metabonomics progress using unlabeled biological fluids [9]–[16] or intact tissues [17]–[19] has made an NMR approach a leading tool in the post-genomics and proteomics era [5], [6]. A combination of SI-labeling with multidimensional NMR analysis opens a new avenue of metabolic phenotyping in higher organisms, and allows complicated metabolic networks to be visualized with ‘birds-eye-view’ graphics technology, whereas classical targeted approaches focus on specific molecules. We have proposed using a coarse-grained view of metabolic pathways as a method of metabolic phenotyping, in which the overall changes in organization among metabolic pathways can be appreciated. Historically, General System Theory (GST) [66] as expounded by Ludwig von Bertalanffy was an early attempt to understand the interactions between natural phenomena which tend to organize large numbers of independent components, like very large numbers of cells, tissues, organs and organ systems. Currently, many researchers are dedicating significant resources to systems biology approaches [67]–[73], with the fundamental principals of GST as their foundation. A coarse-grained representation based on a top-down approach must contain information about organization of the system to shed light on biological changes or interactions. In another words, a complex system like a living organism is essentially dependent on higher-dimensional correlations (*i.e.* organization) among a large number of elements. Coarse-grained representations are widely used in various scientific disciplines [42], [74]–[77], and in this work we have successfully discriminated the patterns of metabolic pathways associated with developmental stages in a model invertebrate.

For convenience, we supposed that PC1 reflected a specific phenomenon, possibly ecdysis, because there was a relatively large difference in PC1 scores before and just after ecdysis (**Fig. 5a**). However, starvation might be a better interpretation of PC1, because silkworms do not eat during ecdysis. Either way, the loading of a metabolite on PC1 was interpreted to reflect the correlation between the change of ^13^C intensities in the metabolite and ecdysis or its associated behaviors. The optimized relative positions of KEGG metabolic pathways based on the total intensities of metabolites within each pathway thus provides a relatively robust coarse-grained pattern of silkworm metabolic activity around ecdysis (**Fig. 5b–d**). The shift from ecdysis to fifth instar resulted in a reduction in ^13^C intensities in negatively correlated pathways (**Fig. 5d**, toward the perimeter, cyan), and in an increase in positively correlated pathways (**Fig. 5d**, in the center, yellow), suggesting that metabolic activities can be detected at the metabolic pathway level as well as at the metabolite level. Simultaneous identification of multiple metabolites, and information about their coexistence within a single pathway can provide clues about the relationship between development or environmental interactions, and the metabolic adjustments that accompany such events. Further, our coarse-grained view may offer information about correlated pathways.

The identified pathways could be classified into two categories and assigned colors, making changes in their activities relatively easy to follow during ecdysis (**Fig. 5b–d**). The color of a pathway is determined by the superimposition of the two weighted colors with green as the intermediate indicator of increasing or decreasing relative ^13^C intensities during ecdysis from day 7 to day 12. These two metabolic categories exhibited weak tendencies of energy and nitrogen metabolism in the insect hemolymph [78]. Nitrogen is a major constituent of many major metabolites, such as L-ornithine, asparagine and GABA, which increased toward day 12 (yellow in **Fig. 5b–d**, see also **Table S3**). These metabolites, along with fumarate and glutamate, are synthesized and catabolized as part of the general urea cycle, and the metabolism of amino groups. Furthermore, GABA, glutamate, malate, oxalacetate, fumarate and glutamine relating to glutamate metabolism also exhibited similar tendencies. Nitrogen metabolism is the common thread between these structurally diverse compounds from assimilation to elimination [78]. The metabolites belonging to metabolic pathways in positive total loadings of PC1, *e.g.* L-ornithine, asparagine, oxalacetic acid, or GABA, which had higher positive loadings, exhibted non-linear (V-shaped) time-dependence of signal intensities in which the intensities all went down at ecdysis (day 7) and up during the fifth instar stage (day 10–12). Related metabolic pathways tended to be organized toward the center rather than in the periphery at day 12 (**Fig. 5d**). A previous report also discussed the strong regulation of nitrogen metabolism (especially re-assimilation of ammonia) during the development of *B. mori* larvae [79]. The silkworm is in a fasting state during metamorphosis (after day 12), so these key metabolites may be stockpiled in the hemolymph in anticipation of nutrient deprivation. On the other hand, 3-phosphoglycerate, acetate, lactate, serine, and pyruvate increased only at day 7, by which time ecdysis (starved state) would be finished. These metabolites are a part of the glycolysis/gluconeogenesis pathways and cysteine metabolism (blue in **Fig. 5b–d**). Since the insect can derive energy from the catabolism of lipids during its starvation periods [80], these metabolite levels might be increased at day 7. Incorporated ^13^C can be highly accumulated in lipid bodies (Nishihara, T., unpublished results). Overall, these physiological changes in silkworm growth were well-characterized by our method.

One of the limitations of applying this method to higher organisms is the ability to label with SI at high concentrations, especially in chemoheterotrophic organisms. However, our experience with SI labeling in a wide range of higher organisms, indicates that invertebrates, and insects in particular, tend to be easily labeled by incorporation of glucose and/or amino acids. Hence omnivores, such as rodents, might become important model organisms in metabolomics as well as in other meta-analytical fields for comparisons with humans. We have recently reported SI labeling in mouse intestine, resulting in the successful *in vivo* detection of the anti-bacterial metabolite reuterin [81].

Another limitation is that there is no direct evidence linking an identified metabolite with a unique pathway if the connection is limited to the methods reported here, because many metabolites are substrates or products of several pathways. Coincident detections of a metabolite in the same pathway, and correlation on the PC1 axis lend some support. Combining SI-labeling techniques can also support them. For example, ^13^C-labeled positions inside a molecule offer a hint about paths of molecular conversions.

Our graphic representation may not be adequate to serve as a quantitative tool for understanding these higher dimensional correlations, but the apparent coarse-grained patterns of increasing and decreasing ^13^C intensities in metabolites and pathways occurred coincidently throughout related metabolic pathways during the period from ecdysis to the fifth instar. This method can thus be used to screen physiological changes in organisms by using coarse-grained views of metabolic pathways in which it would be useful to have an overview of several specific metabolic states.

## Methods

### Chemicals and biological samples

SI-labeled compounds were purchased and fed to *Bombyx mori* fourth instar larvae (Ehimesansyu Co. Ltd., Ehime, Japan) or T87 *Arabidopsis thaliana* cells (RIKEN BioResource Center, Tsukuba, Ibaraki, Japan) to examine chemical shifts (see **Text S1**). T87 cells were incubated and subcultured every 7 days in 20 mL of JPL medium [82] supplemented with 0.5% (v/v) glucose in a 100-mL baffled Erlenmeyer flask on a rotary shaker at 100 rpm and 24°C under a 16-h light / 8-h dark cycle unless otherwise noted. Cells were labeled with [^13^C_6_]glucose as described elsewhere [31]. *B. mori* larvae were grown on 1 g of a semi-synthetic diet medium (Katakura-kogyo Co. Ltd., Hachioji, Japan) supplemented with 200 µL of an aqueous solution containing 2% (w/w) [^13^C_6_]glucose and 1% (w/w) [^13^C,^15^N]amino acids (AA) at 26°C under a 10-h light/14-h dark cycle for 12 days. The hemolymph from SI-labeled silkworms was sampled on days 2, 6, 7, 10, and 12.

### Standard buffer for NMR

Chemical shift reference buffer (100 mM, pH 7.0, 1.0 mM DSS) was made from 1 M aqueous potassium phosphate stock solutions (KH_2_PO_4_ and K_2_HPO_4_), and 2,2-dimethyl-2-silapentane-5-sulfonate (DSS) in deuterium oxide (D_2_O) water.

### NMR extracts

Non-labeled T87 cells were washed twice with water, lyophilized, and ground to powder. 5 mg of the powder was suspended in 600 µL of the standard buffer, heated to 50°C for 5 min, and centrifuged at 10000× *g* for 5 min. 500 µL of supernatant was decanted into a 5-mm ø NMR tube. ^13^C-labeled T87 extracts were prepared essentially as described elsewhere [31]. ^13^C- and ^15^N-labeled silkworm hemolymph was extracted from one or a few silkworms by centrifugation of hemocytes at 4000× *g* for 5 min. 162 µL of each hemolymph sample was added to 18 µL of 1 M potassium phosphate buffer (10% D_2_O, 5 mM DSS), and then transferred to a 5-mm ø Shigemi NMR tube.

### NMR spectroscopy

All HSQC [29] and 3D HCCH-COSY [49] spectra were acquired at 298 K on a Bruker AVANCE DRX 500 (AVANCE DRU 700) NMR spectrometer operating at 500.13 (700.15) MHz and equipped with an ^1^H inverse cryogenic probe with Z-axis gradients (see **Text S1**). Spectra were processed using the NMRPipe software package [83] with window functions, zero-fillings, linear predictions, and polynomial baseline corrections. ^13^C-HSQC peaks were identified by an automated algorithm embedded in the software and refined manually. Peak intensities were determined with peak heights.

### Metabolite chemical shift database

Each purchased standard compound for compiling into the database was measured by NMR under standardized conditions. Chemical shift fluctuations with and without the addition of unlabeled T87 cell extracts from seven different cultures were examined. One of the extracts was from cells grown under a 24-h light (LL) cycle. The pH of samples was determined (model B-212 Horiba pH meter) and ^13^C-HSQC spectra were recorded in standard buffer only (control), standard buffer with non-labeled T87 extract, or in standard buffer with labeled T87 extract. Chemical shift fluctuations were defined as the differences in chemical shifts between labeled and control. The chemical shifts of labeled compounds were derived by comparing labeled and non-labeled spectra. Chemical shift fluctuations for succinate and fumarate in T87 extracts were examined by ^13^C-HSQC and identified by their chemical shift values and visual inspection. Chemical shift fluctuations due to very small differences in buffer preparation (artifacts) were determined by a comparison of control spectra using standardized buffers from different lots but having the same composition. The pH dependence of chemical shifts for 16AA, glucose, succinate, and fumarate was examined by ^13^C-HSQC at seven different pH values in citric acid–phosphate buffer at pH 3; acetate–sodium acetate or succinate buffer at pH 4 and 5; potassium phosphate buffers at pH 6 and 7; and sodium hydroxide–boric acid buffers at pH 8 and 9. Our chemical shift data are continuously updated and are available on the PRIMe website [84] (http://prime.psc.riken.jp/).

### Systematic batch identification of metabolites

The metabolite chemical shift database was implemented with an in-house Java program which allows systematic batch identification of large numbers of metabolites by simply matching the queried observed ^13^C-HSQC peaks with peaks in the implemented metabolite chemical shift database. It also allows aliased chemical shifts. Each of the queried observed peaks was defined as identified or assigned when the chemical shift difference in each dimension between the observed peak and that of the database was less than some tolerance value. Typically, tolerances of 0.03 to 0.04 ppm for ^1^H and 0.3 to 0.4 ppm for ^13^C were used in this study. An identification or assignment was defined as unique if there was only one candidate in the database within the specified tolerances for an observed peak.

### Coarse-grained views of metabolic networks

Sequential ^13^C-HSQC spectra for silkworms in a ^13^C-feeding experiment were obtained twice daily on five different days. The resulting 56 uniquely identified metabolites common throughout these ten spectra were converted to a 56×56 (metabolite–metabolite) correlation matrix in which an element of the matrix was defined as Pearson's coefficient of correlation between the representative intensity of a metabolite and that of any other metabolite (**Fig. 4b**). The representative intensity of a metabolite was defined as the ratio of the summation of the intensities of all of the ^13^C-HSQC peaks for a metabolite to that of DSS, and corresponds to the abundance of ^13^C atoms to the metabolite. Principal Components Analysis (PCA) was performed on the correlation matrix by Eigenvalue decomposition with Mathematica software (Wolfram Research), and the principal component scores and loadings on PC1 and PC2 were determined. Using a simulated annealing technique and 132 KEGG Markup Language files (KGML, version 0.6) obtained from KEGG [47] (http://www.genome.ad.jp/kegg/), we computed coarse-grained representations of metabolic pathways. The simulated annealing algorithm first allocated a total intensity value to each pathway, consisting of the summation of the representative intensities of the identified metabolites in the pathway. The color of a pathway was computed on the basis of total loadings on the PC1 of the pathway (see the Pilot Application section in the Results section). Next, all of the pathways were randomly scattered within the area of the figure, and Metropolis Monte Carlo simulations were sequentially performed by gradually cooling the temperature to calculate a coarse-grained representation of the metabolic pathways. The total energy of the system was defined in terms of the distance of each pathway from the center, overlapped areas between two pathways, and between a pathway and the periphery of the figure (see **Text S1**). The algorithm was designed to minimize overlaps between pathways and to place the pathways that have a larger (or smaller) total intensity toward the center (or outside) (see the Pilot Application section). Since a simulated annealing technique is a heuristic procedure, it was performed 3 times for each sampling stage, resulting in 15 coarse-grained views. Although similar views were obtained at each day stage, we selected one of the views by visual inspection in which the positions of the pathways were maintained according to their colors (**Fig. 5b–d**). Since this layout algorithm only includes general parameters such as the sizes of components or intensities, it does not include any specific parameters dependent on only KEGG database. However, the results can vary slightly if the definition of a metabolic pathway, or a component of a pathway, is slightly different from that used in our method.

## Supporting Information

Text S1 (0.07 MB DOC)

Figure S1 (0.18 MB DOC)

Figure S2 (0.09 MB DOC)

Figure S3 (0.89 MB DOC)

Table S1 (0.11 MB DOC)

Table S2 (0.06 MB DOC)

Table S3 (0.06 MB DOC)

Table S4 (0.19 MB DOC)
