# Correction: Systematic NMR Analysis of Stable Isotope Labeled Metabolite Mixtures in Plant and Animal Systems: Coarse Grained Views of Metabolic Pathways

**DOI:** 10.1371/annotation/55efe5d9-ca11-4f68-ac03-49a31d803161

**Published:** 2009-04-16

**Authors:** Eisuke Chikayama, Michitaka Suto, Takashi Nishihara, Kazuo Shinozaki, Takashi Hirayama, Jun Kikuchi

The incorrect table was published in place of Table S2. The correct table can be viewed here: 
