# Correction: Systematic NMR Analysis of Stable Isotope Labeled Metabolite Mixtures in Plant and Animal Systems: Coarse Grained Views of Metabolic Pathways

**DOI:** 10.1371/annotation/b9b4135b-d76f-4990-b5e4-a70d85acf8e6

**Published:** 2009-06-05

**Authors:** Eisuke Chikayama, Michitaka Suto, Takashi Nishihara, Kazuo Shinozaki, Jun Kikuchi

There are errors in the authorship, author contributions, and funding section. Takashi Hirayama should not have been listed. The correct citation is: Chikayama E, Suto M, Nishihara T, Shinozaki K, Kikuchi J (2008) Systematic NMR Analysis of Stable Isotope Labeled Metabolite Mixtures in Plant and Animal Systems: Coarse Grained Views of Metabolic Pathways. PLoS ONE 3(11): e3805. doi:10.1371/journal.pone.0003805

The correct author contributions are: Conceived and designed the experiments: EC TN KS JK. Performed the experiments: MS TN JK. Analyzed the data: EC. Contributed reagents/materials/analysis tools: EC. Wrote the paper: EC JK.

The correct funding information is: J.K. was supported in part by grants from RIKEN (the President's Special Research Grant), the Japan Science and Technology Agency (CREST to J.K.), Bio-oriented Technology Advancement Institution (BRAIN to J.K.) and the Ministry of Education, Culture, Sports, Science and Technology of Japan (No. 17710191 to J.K.).

