# Supplementary material for: Systematic NMR Analysis of Stable Isotope Labeled Metabolite Mixtures in Plant and Animal Systems: Coarse Grained Views of Metabolic Pathways
Source: PLoS One. 2008 Nov 25;3(11):e3805. doi: 10.1371/journal.pone.0003805 (PMC2583929; doi:10.1371/journal.pone.0003805)
Supplement: Text S1 — (0.07 MB DOC) [file pone.0003805.s001.doc]

Supporting Information Text

**Chemicals.** The ^13^C,^15^N-labeled algal amino acid mixture (16AA, 97.4% ^13^C, 97.9% ^15^N) was purchased from Shoko Co. Ltd., [^13^C_6_]glucose (>98% ^13^C) from Spectra Stable Isotopes, and D_2_O (90%–95% D) from Shoko Co. Ltd. 16AA did not contain asparagine, glutamine, cysteine, or tryptophan.

**NMR acquisition parameters and spectrum analyses.** ^13^C-HSQC spectra used in the HSQC-based metabolite chemical shift database were typically acquired with 1024 points per 16 ppm in the ^1^H dimension, accumulating 16 transients per free induction decay and 128 increments per 76 ppm in the ^13^C dimension on a Bruker DRX 500 or DRU 700. The ^13^C-HSQC spectra used for chemical shift fluctuations were acquired with 1024 points per 12 ppm of ^1^H, 8 transients per free induction decay, and 80 increments per 40 ppm of ^13^C on a Bruker DRU 700. The ^13^C-HSQC spectrum shown in **Fig. 2a** was acquired with 2048 points per 16 ppm of ^1^H, 32 transients per free induction decay, and 160 increments per 40 ppm of ^13^C on a Bruker DRU 700. The ^13^C-HSQC spectrum in **Fig. 2b** was acquired with 1024 points per 16 ppm of ^1^H, 32 transients per free induction decay, and 200 increments per 40 ppm of ^13^C on a Bruker DRU 700. The 3D HCCH-COSY spectrum in **Fig. 3** was acquired with 1024 points per 14 ppm in the ^1^H direct dimension, accumulating 16 transients per free induction decay, 100 increments per 40 ppm in the ^13^C dimension, and 48 increments per 8 ppm in the indirect ^1^H dimension on the DRU 700. The ^13^C-HSQC spectra used to generate **Fig. 4** were acquired with 1024 points per 16 ppm in the ^1^H dimension and 160 increments per 36 ppm in the ^13^C dimension on the DRX 500. To confirm metabolite identification in the HSQC spectrum obtained by 3D HCCH-COSY, all of the detected ^13^C-HSQC peaks were manually examined as follows: (1) ^13^C-HSQC peaks were grouped by metabolite. (2) For each peak within a group, it was determined whether the corresponding plane in the 3D HCCH-COSY spectrum had cross peaks that showed intra-connectivity within the group (metabolite). (3) A peak was defined as confirmed if the peak had intra-connectivity within the group (metabolite) (typically, it had more than two or three connections) without any conflicts with other assignments.

**Pathway color and simulated annealing.** Each pathway was colored using a standard RGB color having an alpha channel (transparency). Our demonstration graphic representation was based on cyan and yellow. The color of a pathway containing identified metabolites, *PW*_i_, was defined as

$\left\{\begin{matrix} PW_{i}(red)=f_{i}\times Cyan(red)+\left(1-f_{i}\right)\times Yellow(red) \\ PW_{i}(green)=f_{i}\times Cyan(green)+\left(1-f_{i}\right)\times Yellow(green) \\ PW_{i}(blue)=f_{i}\times Cyan(blue)+\left(1-f_{i}\right)\times Yellow(blue) \\ PW_{i}(alpha)=f_{i}\times Cyan(alpha)+\left(1-f_{i}\right)\times Yellow(alpha) \end{matrix}\right.$,

where $f_{i}=\frac{p_{i}}{p_{i}-m_{i}}$is the fraction of positive loadings within the pathway; $p_{i}=\sum_{\text{only positive}}^{\text{pathway(i)}} LPC1_{k}$, is the summation of positive loadings within the pathway; $m_{i}=\sum_{\text{only negative}}^{\text{pathway(i)}} LPC1_{k}$, is the summation of negative loadings within the pathway; and *LPC1_k_* is the loading on PC1 of metabolite *k* in pathway *i*. A pathway with no identified metabolites was colored gray.

The total energy of the system, *E*, was defined as

$E=-\sum_{i=1}^{N} \frac{8\times10^{7}I_{i}}{R_{i}}+\sum_{i=1}^{N} \sum_{\begin{matrix} j=1 \\ \left(i\neq j\right) \end{matrix}}^{N} \frac{\delta_{ij}^{2}}{\left(1-\frac{\delta_{ij}}{A_{i}}\right)^{2}\left(1-\frac{\delta_{ij}}{A_{j}}\right)^{2}}+\sum_{i=1}^{N} \varepsilon_{i}$,

where $I_{i}$ is the sum of standardized intensities of metabolites, which are the standardizing values in **Fig. S3**, within a given pathway *i* (= 100 for pathways containing no identified metabolites); $R_{i}$ is the distance of pathway *i* from the center in pixels; *N* is the number of pathways; *δ_ij_* is the area of overlap of pathways *i* and *j* in pixels^2^; *A_i_*, the area of pathway *i* (represented by a rectangle) in pixels^2^; *A_j_*, the area of pathway *j* in pixels^2^; $\varepsilon_{i}=\left\{\begin{matrix} \infty\text{; intersected} \\ \text{0; otherwise} \end{matrix}\right.$ is the energy of pathway *I*, which is infinite for pathways overlapping the periphery of the figure. The initial and final temperatures were 3,000,000 and 1000, respectively. The initial temperature became cooler with every simulation; the temperature for the next simulation was obtained by multiplying the previous temperature by 0.955. The number of steps per simulation at a given temperature was increased with each simulation by multiplying the previous number of steps by 1.02 to obtain the number of steps for the next simulation. The number of steps at the initial temperature was 132 × 132 × 10 = 174240. The first term of the equation is similar to the gravitational potential energy and attracts pathways having larger intensities toward the center, but repels pathways having weaker intensities and no identified metabolites toward the periphery. The coefficient 8 × 10^7^ was optimized by visual inspection of the results of multiple simulations. The second term represents the repulsive energy when two pathways overlap, thus minimizing overlaps. The third term restricts a pathway to the drawing area.
