# Supplementary material for: Systematic NMR Analysis of Stable Isotope Labeled Metabolite Mixtures in Plant and Animal Systems: Coarse Grained Views of Metabolic Pathways
Source: PLoS One. 2008 Nov 25;3(11):e3805. doi: 10.1371/journal.pone.0003805 (PMC2583929; doi:10.1371/journal.pone.0003805)
Supplement: Figure S1 — (0.18 MB DOC) [file pone.0003805.s002.doc]

Supporting Information Figure S1.


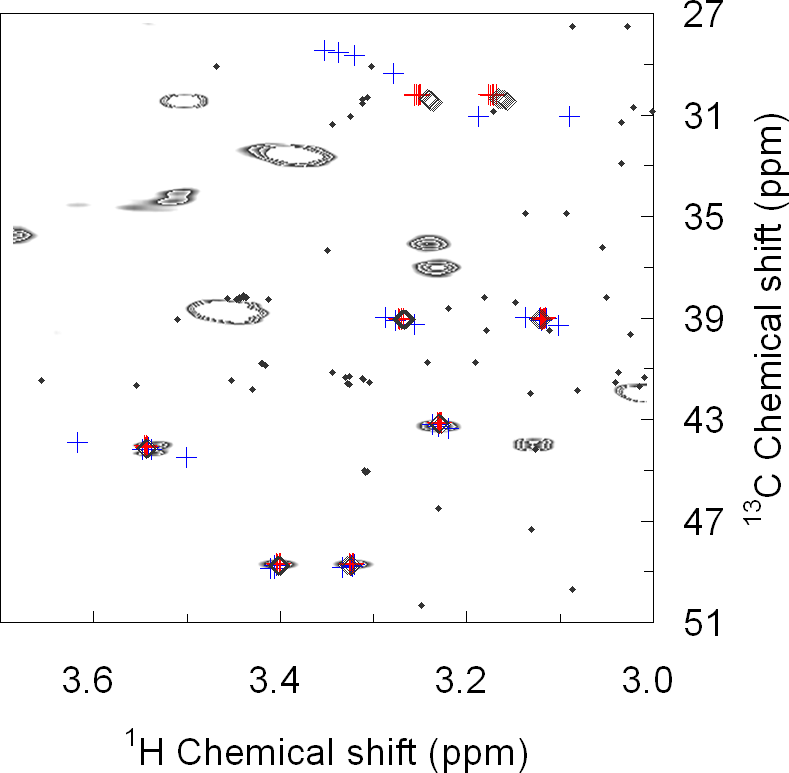


**His β**

**Phe β**

**Arg δ**

**Pro δ**

**Gly α**

**Fig. S1.** The chemical shifts of histidine β and glycine α exhibit large fluctuations in response to pH (blue crosses). Proline δ, arginine δ, and phenylalanine β are not affected. The chemical shifts of crude extracts (red crosses) and samples from different lots of standardized buffers (gray diamonds) are relatively stable. Peaks in the HSQC-based metabolite chemical shift database (gray dots) and the T87 extract raw spectrum are shown for comparison.
