# Supplementary material for: Systematic NMR Analysis of Stable Isotope Labeled Metabolite Mixtures in Plant and Animal Systems: Coarse Grained Views of Metabolic Pathways
Source: PLoS One. 2008 Nov 25;3(11):e3805. doi: 10.1371/journal.pone.0003805 (PMC2583929; doi:10.1371/journal.pone.0003805)
Supplement: Figure S2 — (0.09 MB DOC) [file pone.0003805.s003.doc]

Supporting Information Figure S2.


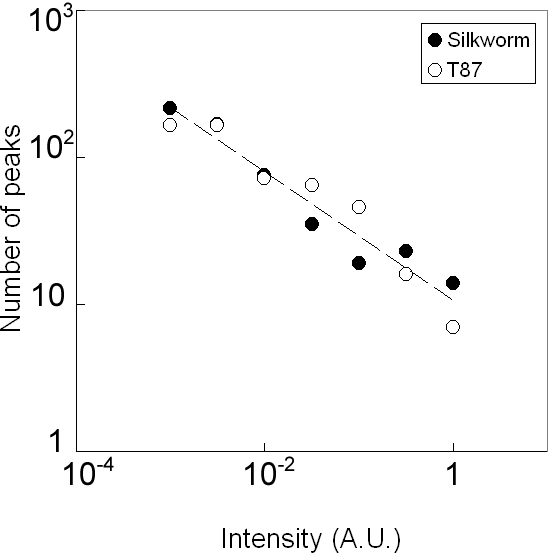


**Fig. S2.** The intensities of observed peaks in silkworm and T87 HSQC spectra (**Fig. 3**) exhibit a power-law relationship with the number of peaks. The intensities were normalized relative to the highest intensity in each spectrum. log(number of peaks) = – 0.44 × log(intensity) + 1.0 (*r*^2^ = 0.93).
