# Supplementary material for: Systematic NMR Analysis of Stable Isotope Labeled Metabolite Mixtures in Plant and Animal Systems: Coarse Grained Views of Metabolic Pathways
Source: PLoS One. 2008 Nov 25;3(11):e3805. doi: 10.1371/journal.pone.0003805 (PMC2583929; doi:10.1371/journal.pone.0003805)
Supplement: Figure S3 — (0.89 MB DOC) [file pone.0003805.s004.doc]

Supporting Information Figure S3.


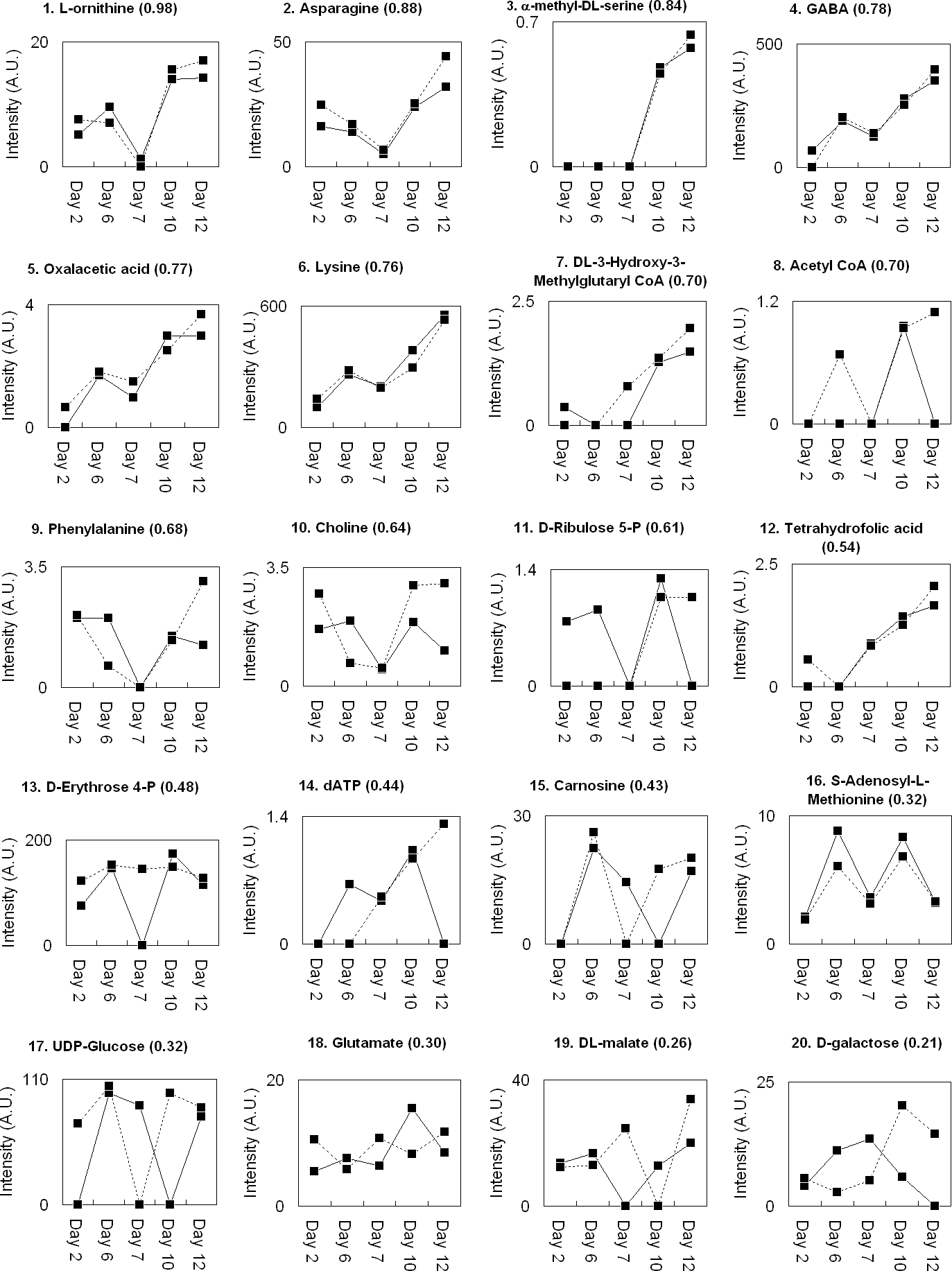


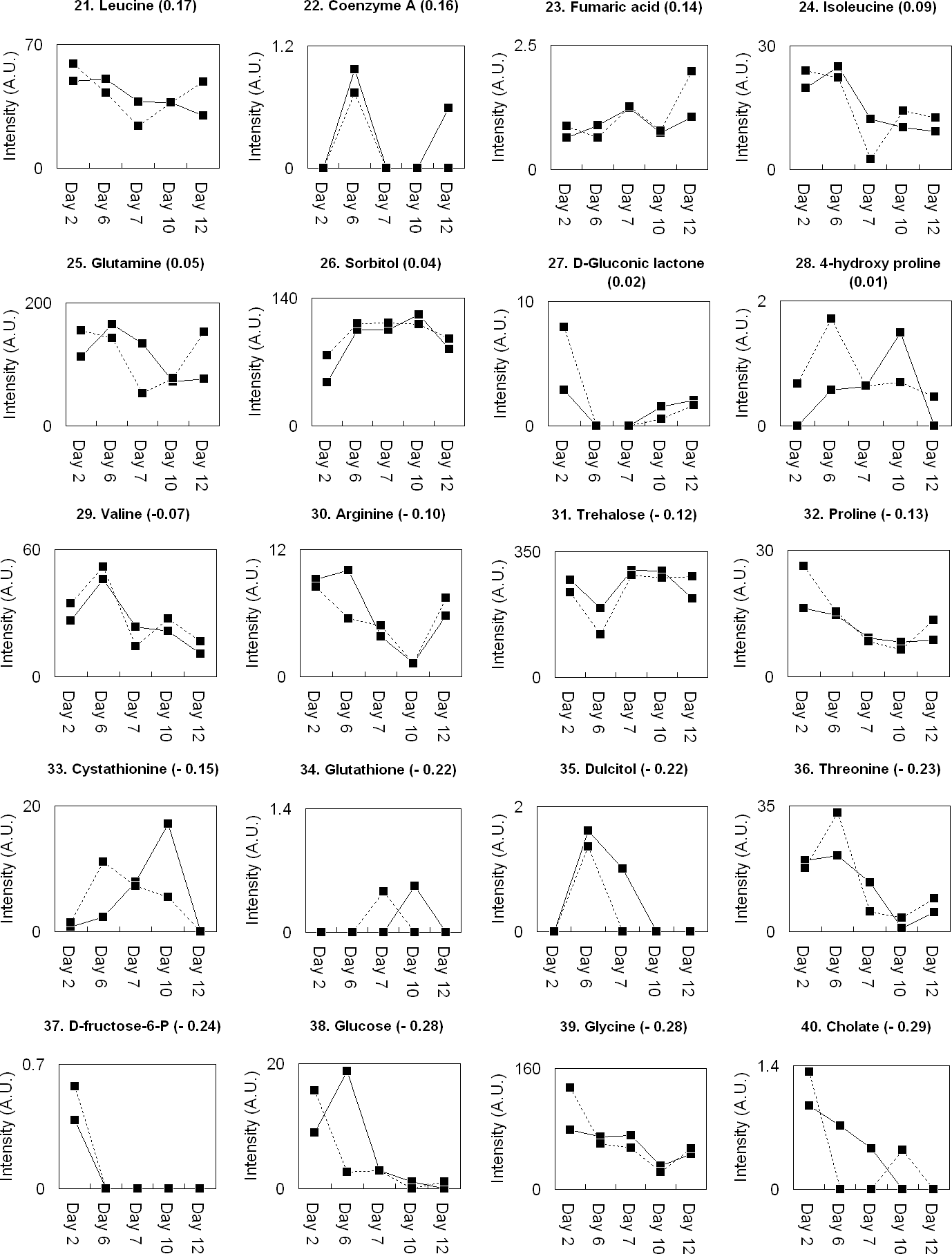


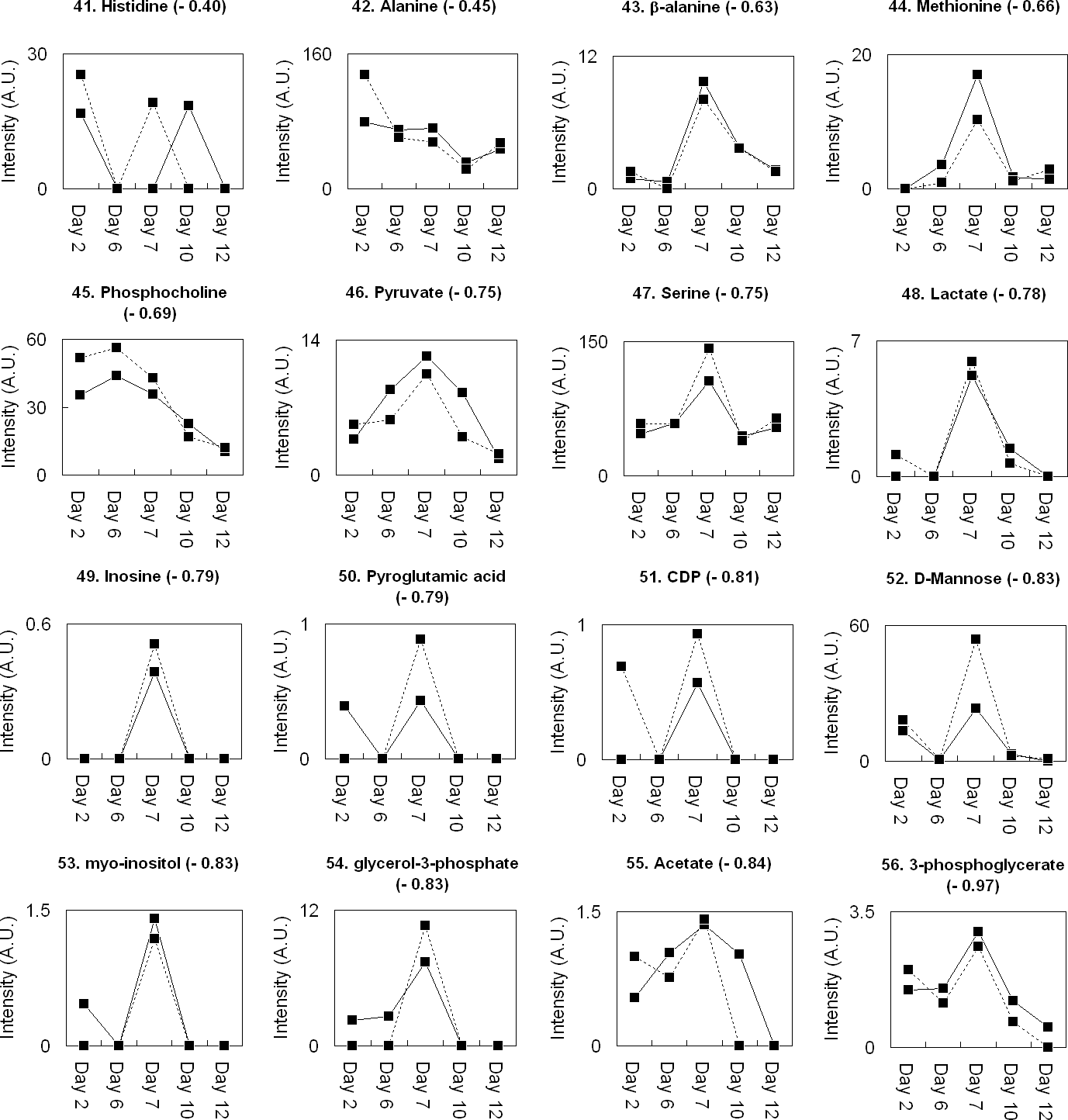


**Fig. S3.** Total peak intensities of the 56 identified candidate metabolites detected in silkworm hemolymph in relation to sampling stage. The intensities were normalized to DSS so that comparisons of intensities between plots would be meaningful. The intensities are zero where the corresponding metabolite was not detectable. The experiments were performed twice (solid and dotted lines). Indices and names correspond to **Table S3**. Values in parentheses denote PC1 loadings.
