# Supplementary material for: Systematic NMR Analysis of Stable Isotope Labeled Metabolite Mixtures in Plant and Animal Systems: Coarse Grained Views of Metabolic Pathways
Source: PLoS One. 2008 Nov 25;3(11):e3805. doi: 10.1371/journal.pone.0003805 (PMC2583929; doi:10.1371/journal.pone.0003805)
Supplement: Table S1 — (0.11 MB DOC) [file pone.0003805.s005.doc]

**Table S1.** Candidate metabolites numbered in **Fig. 2.**

| No. | Metabolite (Silkworm) |
| --- | --- |
| 1 | Phenylalanine |
| 2 | Phenylalanine |
| 3 | Phenylalanine |
| 4 | Carnosine |
| 5 | Carnosine |
| 6 | Histidine |
| 7 | Fumaric acid |
| 8 | Adenosine |
| 9 | 4-hydroxy praline |
| 10 | Glucose |
| 11 | Adenylosuccinic acid |
| 12 | UDP-Glucose |
| 13 | 4-hydroxy praline |
| 14 | Threonine |
| 15 | Phosphocholine |
| 16 | 3-phosphoglycerate |
| 17 | Proline |
| 18 | D-Galactose |
| 19 | Tryptophan |
| 20 | Choline |
| 21 | Tryptophan |
| 22 | D-Erythrose 4-phosphate |
| 23 | Raffinose |
| 24 | Ascorbate |
| 25 | Asparagine |
| 26 | D-Ribose |
| 27 | Dulcitol |
| 28 | Serine |
| 29 | Phenylalanine |
| 30 | Dulcitol |
| 31 | D-Ribulose 5-phosphate |
| 32 | UDP-Glucose |
| 33 | Raffinose |
| 34 | D-Mannose |
| 35 | D-Mannose |
| 36 | Alanine |
| 37 | Dihydroxyacetone phosphate |
| 38 | Gluconic acid lactone |
| 39 | UDP-Glucose |
| 40 | D-Galactose |
| 41 | Leucine |
| 42 | Lipoamide |
| 43 | Isoleucine |
| 44 | Trehalose |
| 45 | Oxalacetic acid |
| 46 | D-Erythrose 4-phosphate |
| 47 | Acetyl-L-carnitine |
| 48 | Phosphocholine |
| 49 | Glycine |
| 50 | Glycine |
| 51 | S-Adenosyl-L-methionine |
| 52 | Choline |
| 53 | Choline |
| 54 | Glucose |
| 55 | Proline |
| 56 | 4-hydroxy praline |
| 57 | Proline |
| 58 | Phenylalanine |
| 59 | Glucose |
| 60 | Lipoamide |
| 61 | Arginine |
| 62 | Phenylalanine |
| 63 | S-Adenosyl-L-methionine |
| 64 | S-Adenosylhomocysteine |
| 65 | S-Adenosylhomocysteine |
| 66 | DSS |
| 67 | 2'-Deoxyguanosine |
| 68 | Beta-NADPH |
| 69 | Cystathionine |
| 70 | Carnosine |
| 71 | Methionine |
| 72 | dATP |
| 73 | Beta-alanine |
| 74 | 4-hydroxy praline |
| 75 | Succinyl coenzyme A |
| 76 | Pyruvate |
| 77 | Proline |
| 78 | Pyruvate |
| 79 | S-Adenosyl-L-methionine |
| 80 | Valine |
| 81 | Glutaric acid |
| 82 | Propionate |
| 83 | 4-hydroxy praline |
| 84 | S-Adenosylhomocysteine |
| 85 | Glutamate |
| 86 | Methionine |
| 87 | Glutamate |
| 88 | Proline |
| 89 | Proline |
| 90 | Isoleucine |
| 91 | Acetate |
| 92 | Lysine |
| 93 | DSS |
| 94 | L-ornithine |
| 95 | Leucine |
| 96 | Lysine |
| 97 | Leucine |
| 98 | Arginine |
| 99 | Cholate |
| 100 | Lysine |
| 101 | Alanine |
| 102 | Isoleucine |
| 103 | Lysine |
| 104 | alpha-Methylserine |
| 105 | Threonine |
| 106 | 3-Hydroxy-3-Methylglutaryl CoA |
| 107 | Isoleucine |
| 108 | Valine |
| 109 | Isoleucine |
| 110 | Leucine |
| 111 | Leucine |
| 112 | Isoleucine |
| 113 | DSS |
| 114 | DSS |
| 115 | Trehalose |
| 116 | Carnosine |
| 117 | D-Erythrose 4-phosphate |
| 118 | Asparagine |
| 119 | Asparagine |

| No. | Metabolite (T87) |
| --- | --- |
| 1 | Adenine |
| 2 | Guanosine |
| 3 | Uridine |
| 4 | Uridine |
| 5 | Tryptophan |
| 6 | Uracil |
| 7 | Phenylalanine |
| 8 | Phenylalanine |
| 9 | Phenylalanine |
| 10 | Carnosine |
| 11 | Carnosine |
| 12 | Tyrosine |
| 13 | Fumaric acid |
| 14 | 2'-Deoxyguanosine |
| 15 | 2'-Deoxycytidine |
| 16 | Inosine |
| 17 | Guanosine |
| 18 | Uridine |
| 19 | Uridine |
| 20 | Uracil |
| 21 | Phosphoenolpyruvate |
| 22 | D-Mannose |
| 23 | Raffinose |
| 24 | D-Ribose |
| 25 | Guanosine |
| 26 | D-Galactose |
| 27 | D-Ribose 5-phosphate |
| 28 | Malate |
| 29 | Thymidine 3',5'-cyclic monophosphate |
| 30 | Guanosine |
| 31 | D-Xylulose |
| 32 | 2'-Deoxyadenosine |
| 33 | Pyroglutamic acid |
| 34 | Uridine |
| 35 | Gluconic acid lactone |
| 36 | Proline |
| 37 | D-Fructose 6-phosphate |
| 38 | Lactate |
| 39 | Cholate |
| 40 | Fructose 1,6-biphosphate |
| 41 | Choline |
| 42 | Gluconic acid lactone |
| 43 | Thymidine |
| 44 | Asparagine |
| 45 | D-Ribose |
| 46 | D-Ribose |
| 47 | Serine |
| 48 | 4-Hydroxy-3-methoxycinnamic acid |
| 49 | D-Ribose |
| 50 | Aspartate |
| 51 | Serine |
| 52 | D-Mannose |
| 53 | Ethanolamine |
| 54 | D-Ribose |
| 55 | Dihydroxyacetone phosphate |
| 56 | Alanine |
| 57 | Glycerol |
| 58 | Leucine |
| 59 | D-Galactose |
| 60 | Dulcitol |
| 61 | D-Xylulose |
| 62 | D-Mannose |
| 63 | S-Adenosyl-L-methionine |
| 64 | D-Erythrose 4-phosphate |
| 65 | Glycine |
| 66 | Dihydroxyacetone phosphate |
| 67 | Choline |
| 68 | Proline |
| 69 | Glucose |
| 70 | Proline |
| 71 | Acetoacetyl coenzyme A |
| 72 | 3-Hydroxy-3-Methylglutaryl CoA |
| 73 | Phenylalanine |
| 74 | Arginine |
| 75 | S-Adenosyl-L-methionine |
| 76 | Phenylalanine |
| 77 | S-Adenosyl-L-methionine |
| 78 | Tyrosine |
| 79 | Creatine |
| 80 | S-Adenosyl-L-methionine |
| 81 | Asparagine |
| 82 | DSS |
| 83 | Asparagine |
| 84 | 2'-Deoxyadenosine |
| 85 | Asparagine |
| 86 | Malate |
| 87 | Methionine |
| 88 | beta-alanine |
| 89 | 2'-Deoxyadenosine |
| 90 | Glutathione |
| 91 | Pyroglutamic acid |
| 92 | Pyroglutamic acid |
| 93 | Glutamine |
| 94 | Proline |
| 95 | Valine |
| 96 | Methionine |
| 97 | Methionine |
| 98 | Glutamate |
| 99 | Methionine |
| 100 | Proline |
| 101 | Glutamate |
| 102 | Pyroglutamic acid |
| 103 | Proline |
| 104 | Isoleucine |
| 105 | Cholate |
| 106 | Lysine |
| 107 | Glutaric acid |
| 108 | DSS |
| 109 | Lysine |
| 110 | Leucine |
| 111 | Arginine |
| 112 | Lysine |
| 113 | Isoleucine |
| 114 | Lysine |
| 115 | Lactate |
| 116 | Threonine |
| 117 | 3-Hydroxy-3-methylglutaryl CoA |
| 118 | Isoleucine |
| 119 | Propionate |
| 120 | Valine |
| 121 | Isoleucine |
| 122 | Leucine |
| 123 | Leucine |
| 124 | Isoleucine |
| 125 | Butyrate |
| 126 | DSS |
| 127 | DSS |
| 128 | Phenylalanine |
| 129 | Uridine |
| 130 | Isoleucine |
