# Supplementary material for: Systematic NMR Analysis of Stable Isotope Labeled Metabolite Mixtures in Plant and Animal Systems: Coarse Grained Views of Metabolic Pathways
Source: PLoS One. 2008 Nov 25;3(11):e3805. doi: 10.1371/journal.pone.0003805 (PMC2583929; doi:10.1371/journal.pone.0003805)
Supplement: Table S2 — (0.06 MB DOC) [file pone.0003805.s006.doc]

**Table S3.** Candidate metabolites identified by ^13^C-HSQC spectra during silkworm development from fourth instar through ecdysis to fifth instar.

| No. | NAME |
| --- | --- |
| 1 | L-ornithine |
| 2 | Asparagine |
| 3 | alpha-methyl-DL-serine |
| 4 | GABA |
| 5 | Oxalacetic acid |
| 6 | Lysine |
| 7 | DL-3-Hydroxy-3-Methylglutaryl CoA |
| 8 | Acetyl CoA |
| 9 | Phenylalanine |
| 10 | Choline |
| 11 | D-Ribulose 5-phosphate |
| 12 | Tetrahydrofolic acid |
| 13 | D-Erythrose 4-phosphate |
| 14 | dATP |
| 15 | Carnosine |
| 16 | S-Adenosyl-L-Methionine |
| 17 | UDP-Glucose |
| 18 | Glutamate |
| 19 | DL-malate |
| 20 | D(+)galactose |
| 21 | Leucine |
| 22 | Coenzyme A |
| 23 | Fumaric acid |
| 24 | Isoleucine |
| 25 | Glutamine |
| 26 | Sorbitol |
| 27 | D-Gluconic lactone |
| 28 | 4-hydroxy praline |
| 29 | Valine |
| 30 | Arginine |
| 31 | Trehalose |
| 32 | Proline |
| 33 | Cystathionine |
| 34 | Glutathione |
| 35 | Dulcitol |
| 36 | Threonine |
| 37 | D-fructose-6phosphate |
| 38 | Glucose |
| 39 | Glycine |
| 40 | Cholate |
| 41 | Histidine |
| 42 | Alanine |
| 43 | beta-alanine |
| 44 | Methionine |
| 45 | PhosphoCholine |
| 46 | Pyruvate |
| 47 | Serine |
| 48 | Lactate |
| 49 | Inosine |
| 50 | Pyroglutamic acid |
| 51 | Cytidine 5-diphosphate(CDP) |
| 52 | D-Mannose |
| 53 | myo-inositol |
| 54 | glycerol-3-phosphate |
| 55 | Acetate |
| 56 | 3-phosphoglycerate |
