# Supplementary material for: Systematic NMR Analysis of Stable Isotope Labeled Metabolite Mixtures in Plant and Animal Systems: Coarse Grained Views of Metabolic Pathways
Source: PLoS One. 2008 Nov 25;3(11):e3805. doi: 10.1371/journal.pone.0003805 (PMC2583929; doi:10.1371/journal.pone.0003805)
Supplement: Table S4 — (0.19 MB DOC) [file pone.0003805.s008.doc]

**Table S4.** Total intensities and loadings for all 132 of the KEGG reference metabolic pathways.

| No. | Pathway Total intensity**^1^** | Day 2 | Day 6 | Day 7 | Day 10 | Day 12 | Total loading  on PC1**^2^** |
| --- | --- | --- | --- | --- | --- | --- | --- |
| 1 | Glycolysis / Gluconeogenesis | 1.66 | 0.38 | 4.19 | -0.90 | -5.32 | -3.15 |
| 2 | Citrate cycle (TCA cycle) | -5.09 | 1.42 | 0.16 | 0.80 | 2.70 | 1.15 |
| 3 | Pentose phosphate pathway | 3.29 | 0.70 | -2.71 | 1.10 | -2.37 | -0.13 |
| 4 | Inositol metabolism | -1.05 | -0.61 | 0.84 | 0.91 | -0.09 | -0.13 |
| 5 | Pentose and glucuronate interconversions | -1.75 | 1.57 | -0.41 | 1.08 | -0.49 | 0.19 |
| 6 | Fructose and mannose metabolism | -0.88 | -0.77 | 3.92 | 0.24 | -2.52 | -1.81 |
| 7 | Galactose metabolism | -7.33 | 1.73 | 4.60 | 6.20 | -5.21 | -0.79 |
| 8 | Ascorbate and aldarate metabolism | -0.51 | 0.29 | 1.46 | -0.01 | -1.23 | -0.75 |
| 9 | Fatty acid biosynthesis | -0.92 | -0.07 | -0.92 | 1.45 | 0.45 | 0.70 |
| 10 | Fatty acid elongation in mitochondria | -0.92 | -0.07 | -0.92 | 1.45 | 0.45 | 0.70 |
| 11 | Fatty acid metabolism | -4.62 | 10.01 | -4.62 | -2.25 | 1.49 | 1.69 |
| 12 | Synthesis and degradation of ketone bodies | -1.63 | -1.03 | -1.36 | 2.24 | 1.77 | 1.41 |
| 13 | Biosynthesis of steroids | -1.22 | -0.67 | 1.02 | 0.77 | 0.09 | -0.04 |
| 14 | Bile acid biosynthesis | 0.97 | -0.04 | 0.81 | -1.61 | -0.14 | -0.57 |
| 15 | Ubiquinone biosynthesis | 0.00 | 0.00 | 0.00 | 0.00 | 0.00 | 0.00 |
| 16 | C21-Steroid hormone metabolism | 0.00 | 0.00 | 0.00 | 0.00 | 0.00 | 0.00 |
| 17 | Androgen and estrogen metabolism | 0.00 | 0.00 | 0.00 | 0.00 | 0.00 | 0.00 |
| 18 | Oxidative phosphorylation | -0.71 | -0.69 | 0.69 | -0.71 | 1.43 | 0.14 |
| 19 | Photosynthesis | 0.00 | 0.00 | 0.00 | 0.00 | 0.00 | 0.00 |
| 20 | Urea cycle and metabolism of amino groups | 0.89 | -0.92 | -2.13 | -0.83 | 2.99 | 1.19 |
| 21 | Purine metabolism | -1.90 | 0.39 | 2.31 | -1.59 | 0.78 | -0.59 |
| 22 | Puromycin biosynthesis | 0.00 | 0.00 | 0.00 | 0.00 | 0.00 | 0.00 |
| 23 | Pyrimidine metabolism | -0.50 | 1.18 | 1.94 | -2.23 | -0.39 | -1.06 |
| 24 | Glutamate metabolism | -4.13 | -1.48 | -0.63 | 0.79 | 5.43 | 2.07 |
| 25 | Alanine and aspartate metabolism | -3.95 | -0.47 | 0.34 | -0.70 | 4.78 | 1.36 |
| 26 | Tetracycline biosynthesis | -0.95 | -0.53 | -2.18 | 1.77 | 1.90 | 1.58 |
| 27 | Glycine, serine and threonine metabolism | -0.25 | 0.80 | 4.38 | -2.82 | -2.11 | -2.48 |
| 28 | Methionine metabolism | -2.21 | 0.49 | 3.00 | -0.14 | -1.14 | -1.09 |
| 29 | Cysteine metabolism | -0.16 | -0.62 | 4.22 | -0.82 | -2.63 | -2.17 |
| 30 | Valine, leucine and isoleucine degradation | 0.94 | 2.28 | -4.26 | 1.14 | -0.10 | 1.59 |
| 31 | Valine, leucine and isoleucine biosynthesis | 2.07 | 3.60 | -1.44 | -1.12 | -3.11 | -0.56 |
| 32 | Lysine biosynthesis | -2.00 | -0.21 | -1.51 | 1.73 | 1.99 | 1.47 |
| 33 | Lysine degradation | -2.74 | -0.18 | -0.33 | 0.50 | 2.74 | 1.18 |
| 34 | Penicillins and cephalosporins biosynthesis | 0.24 | 1.59 | -0.61 | -0.21 | -1.00 | -0.07 |
| 35 | Arginine and proline metabolism | -2.80 | 1.45 | -1.83 | 1.73 | 1.44 | 1.56 |
| 36 | Clavulanic acid biosynthesis | 1.02 | 0.67 | -0.48 | -1.48 | 0.27 | -0.10 |
| 37 | Histidine metabolism | -0.21 | -0.81 | -0.55 | 1.24 | 0.32 | 0.32 |
| 38 | Tyrosine metabolism | -2.94 | -0.52 | 5.76 | -1.46 | -0.84 | -1.96 |
| 39 | DDT degradation | 0.00 | 0.00 | 0.00 | 0.00 | 0.00 | 0.00 |
| 40 | Phenylalanine metabolism | -0.45 | -0.49 | 0.53 | -0.68 | 1.08 | 0.07 |
| 41 | gamma-Hexachlorocyclohexane degradation | 0.00 | 0.00 | 0.00 | 0.00 | 0.00 | 0.00 |
| 42 | Benzoate degradation via hydroxylation | -3.23 | 0.40 | 1.47 | 2.16 | -0.80 | -0.02 |
| 43 | Bisphenol A degradation | 0.00 | 0.00 | 0.00 | 0.00 | 0.00 | 0.00 |
| 44 | Tryptophan metabolism | -2.22 | -0.17 | -1.46 | 2.19 | 1.66 | 1.47 |
| 45 | Phenylalanine, tyrosine and tryptophan biosynthesis | 0.18 | 0.70 | -2.95 | 1.16 | 0.91 | 1.16 |
| 46 | Novobiocin biosynthesis | 1.53 | 0.42 | -0.71 | -0.96 | -0.28 | -0.13 |
| 47 | beta-Alanine metabolism | -2.48 | -0.55 | -0.06 | 1.95 | 1.14 | 0.88 |
| 48 | Taurine and hypotaurine metabolism | 0.21 | 0.67 | 1.89 | -0.17 | -2.60 | -1.33 |
| 49 | Aminophosphonate metabolism | 0.11 | 0.37 | 1.32 | -0.39 | -1.40 | -0.84 |
| 50 | Selenoamino acid metabolism | 1.64 | 0.45 | 1.34 | -1.61 | -1.82 | -1.29 |
| 51 | Cyanoamino acid metabolism | -1.23 | -0.69 | 1.67 | -1.69 | 1.95 | -0.16 |
| 52 | D-Glutamine and D-glutamate metabolism | 0.13 | 0.07 | -0.92 | 0.16 | 0.56 | 0.35 |
| 53 | D-Arginine and D-ornithine metabolism | 0.57 | 0.54 | -1.85 | -0.58 | 1.32 | 0.88 |
| 54 | D-Alanine metabolism | 1.02 | 0.37 | 1.49 | -1.23 | -1.65 | -1.20 |
| 55 | Glutathione metabolism | -4.03 | -3.71 | 3.63 | 2.44 | 1.68 | -0.29 |
| 56 | Starch and sucrose metabolism | 3.51 | -0.54 | -0.38 | -1.19 | -1.39 | -0.71 |
| 57 | N-Glycan biosynthesis | 0.00 | 0.00 | 0.00 | 0.00 | 0.00 | 0.00 |
| 58 | O-Glycan biosynthesis | 0.00 | 0.00 | 0.00 | 0.00 | 0.00 | 0.00 |
| 59 | Nucleotide sugars metabolism | -0.95 | 1.40 | -0.67 | -0.47 | 0.69 | 0.32 |
| 60 | Streptomycin biosynthesis | -0.13 | -0.54 | 1.76 | -0.54 | -0.54 | -0.83 |
| 61 | Biosynthesis of 12-, 14- and 16-membered macrolides | -0.92 | -0.07 | -0.92 | 1.45 | 0.45 | 0.70 |
| 62 | Polyketide sugar unit biosynthesis | 0.00 | 0.00 | 0.00 | 0.00 | 0.00 | 0.00 |
| 63 | Aminosugars metabolism | 0.00 | 0.00 | 0.00 | 0.00 | 0.00 | 0.00 |
| 64 | Glycosaminoglycan degradation | 0.00 | 0.00 | 0.00 | 0.00 | 0.00 | 0.00 |
| 65 | Chondroitin sulfate biosynthesis | 0.00 | 0.00 | 0.00 | 0.00 | 0.00 | 0.00 |
| 66 | Heparan sulfate biosynthesis | 0.11 | 0.37 | 1.32 | -0.39 | -1.40 | -0.84 |
| 67 | Lipopolysaccharide biosynthesis | 0.00 | 0.00 | 0.00 | 0.00 | 0.00 | 0.00 |
| 68 | Peptidoglycan biosynthesis | 0.00 | 0.00 | 0.00 | 0.00 | 0.00 | 0.00 |
| 69 | Glycerolipid metabolism | -0.91 | 1.05 | 2.56 | -1.57 | -1.13 | -1.48 |
| 70 | Inositol phosphate metabolism | -0.13 | -0.54 | 1.76 | -0.54 | -0.54 | -0.83 |
| 71 | Glycosylphosphatidylinositol(GPI)-anchor biosynthesis | 0.00 | 0.00 | 0.00 | 0.00 | 0.00 | 0.00 |
| 72 | Glycosylphosphatidylinositol(GPI)-anchor biosynthesis | 0.00 | 0.00 | 0.00 | 0.00 | 0.00 | 0.00 |
| 73 | Glycerophospholipid metabolism | 0.40 | -0.24 | 4.18 | -1.66 | -2.67 | -2.53 |
| 74 | Ether lipid metabolism | 0.00 | 0.00 | 0.00 | 0.00 | 0.00 | 0.00 |
| 75 | Arachidonic acid metabolism | 0.00 | 0.00 | 0.00 | 0.00 | 0.00 | 0.00 |
| 76 | Linoleic acid metabolism | 0.00 | 0.00 | 0.00 | 0.00 | 0.00 | 0.00 |
| 77 | Sphingolipid metabolism | -0.45 | -0.26 | 1.75 | -0.78 | -0.25 | -0.76 |
| 78 | Glycosphingolipid biosynthesis - lactoseries | 0.00 | 0.00 | 0.00 | 0.00 | 0.00 | 0.00 |
| 79 | Glycosphingolipid biosynthesis - neo-lactoseries | 0.00 | 0.00 | 0.00 | 0.00 | 0.00 | 0.00 |
| 80 | Glycosphingolipid biosynthesis - globoseries | 0.00 | 0.00 | 0.00 | 0.00 | 0.00 | 0.00 |
| 81 | Glycosphingolipid biosynthesis - ganglioseries | 0.00 | 0.00 | 0.00 | 0.00 | 0.00 | 0.00 |
| 82 | Pyruvate metabolism | -3.22 | -0.11 | 2.77 | 0.52 | 0.04 | -0.64 |
| 83 | Biphenyl degradation | -0.51 | 0.29 | 1.46 | -0.01 | -1.23 | -0.75 |
| 84 | Toluene and xylene degradation | -0.90 | 0.95 | 4.24 | -0.42 | -3.87 | -2.33 |
| 85 | 2,4-Dichlorobenzoate degradation | 0.00 | 0.00 | 0.00 | 0.00 | 0.00 | 0.00 |
| 86 | 1- and 2-Methylnaphthalene degradation | -1.52 | 0.87 | 4.39 | -0.04 | -3.70 | -2.24 |
| 87 | Tetrachloroethene degradation | -0.92 | -0.07 | -0.92 | 1.45 | 0.45 | 0.70 |
| 88 | Nitrobenzene degradation | -0.51 | 0.29 | 1.46 | -0.01 | -1.23 | -0.75 |
| 89 | 1,4-Dichlorobenzene degradation | -0.51 | 0.29 | 1.46 | -0.01 | -1.23 | -0.75 |
| 90 | Fluorene degradation | -0.51 | 0.29 | 1.46 | -0.01 | -1.23 | -0.75 |
| 91 | Carbazole degradation | -0.51 | 0.29 | 1.46 | -0.01 | -1.23 | -0.75 |
| 92 | Glyoxylate and dicarboxylate metabolism | -3.91 | -0.06 | 0.62 | 1.30 | 2.05 | 0.79 |
| 93 | 1,2-Dichloroethane degradation | 0.00 | 0.00 | 0.00 | 0.00 | 0.00 | 0.00 |
| 94 | Benzoate degradation via CoA ligation | -1.63 | -0.76 | -0.23 | 0.75 | 1.88 | 0.84 |
| 95 | Propanoate metabolism | -2.66 | 1.13 | 1.38 | 2.34 | -2.19 | -0.45 |
| 96 | 3-Chloroacrylic acid degradation | 0.00 | 0.00 | 0.00 | 0.00 | 0.00 | 0.00 |
| 97 | Ethylbenzene degradation | -1.42 | 0.22 | 0.55 | 1.44 | -0.78 | -0.04 |
| 98 | Styrene degradation | -1.59 | -1.02 | 3.91 | -0.88 | -0.42 | -1.39 |
| 99 | Butanoate metabolism | -4.64 | -2.66 | 0.01 | 3.45 | 3.84 | 1.87 |
| 100 | C5-Branched dibasic acid metabolism | -0.90 | -0.53 | 2.52 | 1.01 | -2.11 | -1.29 |
| 101 | One carbon pool by folate | -0.78 | -1.14 | -0.01 | 0.63 | 1.30 | 0.54 |
| 102 | Methane metabolism | -2.40 | -0.42 | 0.81 | 1.01 | 1.00 | 0.28 |
| 103 | Carbon fixation | -4.63 | 0.71 | -0.20 | 0.10 | 4.02 | 1.07 |
| 104 | Reductive carboxylate cycle (CO2 fixation) | -2.03 | -0.11 | 1.72 | -1.25 | 1.66 | -0.16 |
| 105 | Thiamine metabolism | -0.51 | 0.29 | 1.46 | -0.01 | -1.23 | -0.75 |
| 106 | Riboflavin metabolism | -0.29 | -0.12 | -1.20 | 1.57 | 0.05 | 0.61 |
| 107 | Vitamin B6 metabolism | -1.10 | 1.07 | 0.13 | 1.10 | -1.21 | -0.26 |
| 108 | Nicotinate and nicotinamide metabolism | -0.71 | -0.69 | 0.69 | -0.71 | 1.43 | 0.14 |
| 109 | Pantothenate and CoA biosynthesis | -1.44 | 2.73 | 1.90 | -0.69 | -2.51 | -1.28 |
| 110 | Biotin metabolism | -1.08 | -0.14 | -0.59 | 0.27 | 1.54 | 0.76 |
| 111 | Folate biosynthesis | -0.78 | -1.14 | -0.01 | 0.63 | 1.30 | 0.54 |
| 112 | Atrazine degradation | 0.00 | 0.00 | 0.00 | 0.00 | 0.00 | 0.00 |
| 113 | Retinol metabolism | 0.00 | 0.00 | 0.00 | 0.00 | 0.00 | 0.00 |
| 114 | Porphyrin and chlorophyll metabolism | 0.08 | 0.24 | -0.61 | 0.34 | -0.05 | 0.06 |
| 115 | Terpenoid biosynthesis | 0.00 | 0.00 | 0.00 | 0.00 | 0.00 | 0.00 |
| 116 | Indole and ipecac alkaloid biosynthesis | 1.22 | 0.93 | -0.44 | -0.85 | -0.86 | -0.28 |
| 117 | Monoterpenoid biosynthesis | 0.00 | 0.00 | 0.00 | 0.00 | 0.00 | 0.00 |
| 118 | Limonene and pinene degradation | 0.00 | 0.00 | 0.00 | 0.00 | 0.00 | 0.00 |
| 119 | Diterpenoid biosynthesis | 0.00 | 0.00 | 0.00 | 0.00 | 0.00 | 0.00 |
| 120 | Nitrogen metabolism | -0.64 | -0.36 | -1.00 | -0.75 | 2.75 | 0.94 |
| 121 | Sulfur metabolism | -1.25 | 0.41 | 3.53 | 0.09 | -2.78 | -1.74 |
| 122 | Caprolactam degradation | 0.00 | 0.00 | 0.00 | 0.00 | 0.00 | 0.00 |
| 123 | Stilbene, coumarine and lignin biosynthesis | 0.00 | 0.00 | 0.00 | 0.00 | 0.00 | 0.00 |
| 124 | Flavonoid biosynthesis | 0.00 | 0.00 | 0.00 | 0.00 | 0.00 | 0.00 |
| 125 | Alkaloid biosynthesis I | 0.00 | 0.00 | 0.00 | 0.00 | 0.00 | 0.00 |
| 126 | Alkaloid biosynthesis II | 0.62 | 0.96 | -3.30 | -0.09 | 1.82 | 1.53 |
| 127 | Metabolism of xenobiotics by cytochrome P450 | 0.00 | 0.00 | 0.00 | 0.00 | 0.00 | 0.00 |
| 128 | Biosynthesis of ansamycins | -0.95 | 1.40 | -0.67 | -0.47 | 0.69 | 0.32 |
| 129 | Biosynthesis of siderophore group nonribosomal peptides | 0.00 | 0.00 | 0.00 | 0.00 | 0.00 | 0.00 |
| 130 | Biosynthesis of vancomycin group antibiotics | 0.00 | 0.00 | 0.00 | 0.00 | 0.00 | 0.00 |
| 131 | Biosynthesis of type II polyketide backbone | 0.00 | 0.00 | 0.00 | 0.00 | 0.00 | 0.00 |
| 132 | Biosynthesis of type II polyketide products | 0.00 | 0.00 | 0.00 | 0.00 | 0.00 | 0.00 |

^1^ Total intensity at days 2, 6, 7, 10, and 12 is defined as the sum of standardized peak intensities for metabolites included in a pathway (see Fig. S3 for intensities before standardization).

^2^ Total loading on PC1 is defined as the sum of loadings on PC1 for metabolites included in a pathway (Fig. 4c).
