# Supplementary material for: Correction: Systematic NMR Analysis of Stable Isotope Labeled Metabolite Mixtures in Plant and Animal Systems: Coarse Grained Views of Metabolic Pathways
Source: PLoS One. 2009 Apr 16;4(4):10.1371/annotation/55efe5d9-ca11-4f68-ac03-49a31d803161. doi: 10.1371/annotation/55efe5d9-ca11-4f68-ac03-49a31d803161 (PMC2674785; doi:10.1371/annotation/55efe5d9-ca11-4f68-ac03-49a31d803161)
Supplement: Supplementary file 1 [file pone.55efe5d9-ca11-4f68-ac03-49a31d803161.s001.doc]

**Table S2.** HSQC peaks identified with high reliability in T87 extract**s** (**Fig. 2b**) from 3D HCCH-COSY spectra (**Fig. 3**).

| Atom (C-H) | 1H (ppm) | 13C (ppm) |
| --- | --- | --- |
| Adenosine | 4.416 | 73.308 |
| Adenosine | 4.288 | 88.605 |
| Arg Ca | 3.761 | 57.045 |
| Arg Cb | 1.711 | 66.626 |
| Arg Cb | 1.641 | 66.566 |
| Arg Cc | 1.909 | 70.214 |
| Arg Cd | 3.233 | 83.180 |
| Asn Ca | 3.998 | 53.928 |
| Asn Cb | 2.941 | 77.174 |
| Asn Cb | 2.860 | 77.162 |
| choline (CH2) | 4.053 | 58.338 |
| choline (CH2) | 3.511 | 70.088 |
| choline (CH3) | 3.193 | 56.549 |
| D(-)Ribose | 3.519 | 73.814 |
| D(-)Ribose | 4.912 | 56.635 |
| D(+)galactose | 3.698 | 77.869 |
| D(+)galactose | 5.222 | 54.794 |
| D-Gluconic lactone | 4.126 | 76.788 |
| D-Gluconic lactone | 4.026 | 73.628 |
| D-Gluconic lactone | 3.814 | 65.197 |
| D-Gluconic lactone | 3.751 | 75.304 |
| D-Gluconic lactone | 3.755 | 73.792 |
| D-Gluconic lactone | 3.649 | 65.391 |
| DL-malate | 4.297 | 72.959 |
| DL-malate | 2.667 | 85.198 |
| DL-malate | 2.354 | 85.195 |
| GABA | 3.000 | 81.858 |
| GABA | 2.285 | 77.008 |
| GABA | 1.887 | 66.226 |
| Gln Ca | 3.757 | 57.055 |
| Gln Cb | 2.434 | 73.489 |
| Gln Cc | 2.134 | 68.926 |
| Glucose (H1) | 5.222 | 54.794 |
| Glucose (H1') | 4.637 | 58.768 |
| Glucose (H2') | 3.894 | 63.570 |
| Glucose (H3) | 3.462 | 78.583 |
| Glucose (H3') | 3.523 | 74.088 |
| Glucose (H4') | 3.712 | 63.591 |
| Glucose (H5) | 3.235 | 76.777 |
| Glucose (H5') | 3.390 | 72.283 |
| glycerol (CH) | 3.756 | 74.853 |
| glycerol (CH2) | 3.550 | 65.088 |
| lactate (CH) | 4.107 | 71.189 |
| lactate (CH3) | 1.321 | 62.843 |
| Leu Ca | 3.727 | 56.077 |
| Leu Cb | 1.701 | 82.460 |
| Leu Cc | 1.711 | 66.626 |
| Leu Cd | 0.956 | 64.787 |
| Leu Cd | 0.946 | 63.510 |
| Lys Ca | 3.761 | 57.045 |
| Lys Cb | 1.898 | 72.551 |
| Lys Cd | 1.714 | 69.115 |
| Lys Ce | 3.000 | 81.858 |
| Lys Cg | 1.435 | 64.104 |
| Met Cb | 2.193 | 72.342 |
| Met Cb | 2.127 | 72.322 |
| Met Cc | 2.630 | 71.562 |
| Phe Ca | 3.986 | 58.703 |
| Phe Cb | 3.273 | 79.017 |
| Phe Cb | 3.117 | 79.019 |
| Pro Ca | 4.122 | 63.890 |
| Pro Cb | 2.340 | 71.710 |
| Pro Cb | 2.059 | 71.700 |
| Pro Cc | 1.998 | 66.452 |
| Pro Cd | 3.408 | 88.738 |
| Pro Cd | 3.326 | 88.734 |
| pyroglutamic acid | 4.167 | 61.043 |
| pyroglutamic acid | 2.502 | 67.984 |
| pyroglutamic acid | 2.019 | 68.009 |
| Ser Ca | 3.836 | 59.076 |
| Ser Cb | 3.956 | 62.838 |
| Thr Ca | 3.587 | 63.104 |
| Thr Cb | 4.246 | 68.611 |
| Thr Cc | 1.320 | 62.165 |
| Uracil | 5.788 | 63.707 |
| Uridine | 4.342 | 76.331 |
| Uridine | 4.216 | 72.073 |
| Uridine | 4.122 | 86.882 |
| Uridine | 3.896 | 63.271 |
| Uridine | 5.907 | 52.122 |
| Uridine | 5.885 | 64.926 |
| Uridine | 5.904 | 51.829 |
| Uridine | 7.871 | 64.716 |
| Uridine | 7.871 | 64.277 |
| Val Ca | 3.593 | 63.098 |
| Val Cb | 2.267 | 71.772 |
| Val Cc | 1.032 | 60.693 |
| Val Cc | 0.98 | 59.296 |
